# FlhG Cooperates With the Cell Cycle Regulator GpsB to Confine Peritrichous Flagella in *B. subtilis*

**DOI:** 10.1111/mmi.15375

**Published:** 2025-05-19

**Authors:** Anita Dornes, Patrica Bedrunka, Benjamin Pillet, Dieter Kressler, Thomas Heimerl, Jan Pané‐Farré, Gert Bange

**Affiliations:** ^1^ Center for Synthetic Microbiology (SYNMIKRO) and Department of Chemistry Philipps‐University Marburg Marburg Germany; ^2^ Department of Biology University of Fribourg Fribourg Switzerland; ^3^ Max Planck Institute for Terrestrial Microbiology Molecular Physiology of Microbes Marburg Germany

## Abstract

Number and arrangement of flagella, the bacterial locomotion organelles, are species‐specific and serve as key taxonomic markers. The FlhG ATPase (also: YlxH, FleN), along with FlhF, plays pivotal roles in determining flagellation patterns. In *Bacillus subtilis* , FlhG and FlhF govern the spatial arrangement of peritrichous flagella. FlhG aids in flagellar assembly by interacting with the flagellar C‐ring protein FliY, yet the molecular implications of this interaction have been unclear. Our study reveals that the ATP‐dependent FlhG homodimer interacts with the C‐terminal domain of GpsB, a cell cycle regulator, which recruits the peptidoglycan synthase PBP1 (also: *ponA*) to sites of cell wall elongation. A deletion of *gpsB* leads to dysregulation of the flagellation pattern mimicking the effects of a *flhG* deletion strain. The finding that GpsB can interact simultaneously with FlhG and PBP1, combined with the observation that GpsB and FliY can simultaneously interact with FlhG, strongly argues for a model in which FlhG confines flagella biosynthesis to regions of active cell wall biosynthesis. Thus, the FlhG‐GpsB interaction appears to enable the locally restrained stimulation of the GTPase FlhF, known for its role to localize flagella in various bacterial species.

## Graphical abstract

FlhG (orange, with activator helix in green) regulates peritrichous flagellation in *Bacillus subtilis* by interacting with the C‐ring protein FliY (gray, N‐terminus in red) and the cell wall regulator GpsB (N‐terminus in blue, C‐terminus in red). GpsB recruits PBP1 for peptidoglycan synthesis, linking flagellar assembly to cell wall biosynthesis. The GpsB– FlhG interaction thus serves to synchronize the elongation of cells with the orderly development of the flagellum.

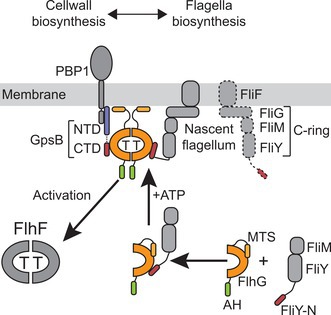

## 1 Introduction

Flagella are molecular machines of motility of bacteria, and their architecture is highly conserved, encompassing components such as the cytoplasmic C‐ring, basal body, rod, and exterior hook and filament structures (Chevance and Hughes 2008). Despite this conservation, bacterial species exhibit variations in the number and arrangement of flagella, known as their flagellation pattern (Schuhmacher, Thormann, et al. 2015; Kazmierczak and Hendrixson 2013). However, the mechanisms governing the precise establishment of these flagellation patterns during each cell division remain poorly understood.

The protein FlhG, also identified as YlxH, MinD2, FleN, or MotR, plays an essential role in determining the correct flagellation pattern in polar‐ (Kusumoto et al. 2008; Dasgupta et al. 2000; Campos‐Garcia et al. 2000), lophotrichous‐ (van Amsterdam and van der Ende 2004), amphitrichous‐ (Balaban and Hendrixson 2011), and peritrichous‐flagellated bacteria (Guttenplan and Kearns 2013; Guttenplan et al. 2013). Deletion of *flhG* in polar‐flagellated bacteria results in hyper‐flagellation and impaired motility (Kusumoto et al. 2008; Dasgupta et al. 2000; Campos‐Garcia et al. 2000). In amphitrichous‐flagellated *Campylobacter jejuni* , approximately 40% of Δ*flhG* cells exhibit multiple flagella at one pole, leading to motility impairment (Balaban and Hendrixson 2011; Gulbronson et al. 2016). The peritrichous‐flagellated *Bacillus subtilis* cell displays around 26 flagellar basal bodies symmetrically arranged in a gridlike pattern on the cell surface, with a characteristic absence of flagella at the cell pole (Guttenplan et al. 2013). While deletion of the gene encoding FlhG in *B. subtilis* does not cause swimming or swarming defects, it results in the appearance of multiple flagella in tufts from specific loci on the cell, resulting from a clustering of flagellar basal bodies (Guttenplan et al. 2013).

FlhG closely resembles the ATPase MinD in structure, and *akin* to MinD, it forms homodimers dependent on ATP (Schuhmacher, Rossmann, Dempwolff, et al. 2015; Chanchal and Banerjee 2017). Additionally, these homodimers can associate with the membrane through a membrane‐targeting sequence located at the protein's C‐terminus (Altegoer et al. 2014). FlhG collaborates with the signal recognition particle (SRP)‐GTPase FlhF in order to ensure the correct localization and number of flagella (Kusumoto et al. 2008; Bange et al. 2007; Bange et al. 2011; Rossmann et al. 2015). Importantly, FlhG interacts with the flagellar C‐ring proteins FliY (aka FliN) in *B. subtilis* in a nucleotide‐independent manner, activating FliY to assemble with its partnering protein FliM into the flagellar C‐ring (Schuhmacher, Thormann, et al. 2015; Schuhmacher, Rossmann, Dempwolff, et al. 2015; Blagotinsek et al. 2020). However, it is unclear how FlhG executes its function in ensuring the correct positioning of bacterial flagella in *B. subtilis* . Thus, we set out to fill this gap.

Our study shows that FlhG interacts with GpsB in an ATP‐dependent manner. GpsB is part of the late *B. subtilis* cell division machinery (Halbedel and Lewis 2019; Tavares et al. 2008). Recently, GpsB has also been shown to act as an adaptor protein, which connects a major class A peptidoglycan synthase with other cell wall and cell cycle proteins, and to cell shape determinants (Cleverley et al. 2016; Cleverley et al. 2019; Rismondo et al. 2016). Thus, our study shows that FlhG communicates with the cell division/elongation machinery of *B. subtilis* in order to enable the correct distance between the laterally arranged flagella.

## 2 Materials and Methods

### 2.1 Strains and Plasmids

All strains and plasmids used in this study are listed in Tables S2 and S3, respectively. Primers are listed in Table S4. The following genes and/or corresponding up‐ and downstream regions were amplified from genomic *B. subtilis* NCIB 3610 and *G. thermodenitrificans* NG80‐2 DNA by PCR using Phusion polymerase and were used for the construction of plasmids for different applications: *B. subtilis* NCIB 3610: *flhG* (BSU16410), *gpsB* (BSU22180), *minD* (BSU27990), *flgE* (BSU16290), *flhF* (BSU16400); *G. thermodenitrificans* NG80‐2: *flhG* (GTNG_1094), *gpsB* (GTNG_1390), *ponA* (GTNG_2102), *fliY* (GTNG_1084), *fliM* (GTNG_1083), *flhF* (GTNG_1092).

For protein production in *E. coli* , the PCR products were introduced into pET24d (Novagen) to generate His‐tagged proteins and into pGAT3 (Novagen) for GST‐tagged proteins. pET24d and pGAT3 plasmids were modified for modular cloning via BsaI restriction sites. *B. subtilis* strains with deletions in or mutations of *flhG*, *flgE, hag, gpsB*, and *ponA* were generated using the pMAD system and previously established protocols (Arnaud et al. 2004). Briefly, PCR‐amplified DNA fragments covering 800 base pairs from the flanking regions up‐ and downstream of the target gene were cloned into the BamHI and EcoRI restriction sites of pMAD. To introduce point mutations, two overlapping fragments of the target gene were amplified using primers that introduced the desired mutation in the overlapping region.

The pMAD construct introducing the T123C substitution into FlgE was transformed into *B. subtilis* NCIB 3610, generating strain PB11. This strain served as the genetic background for the construction of strains with deletions in *flhG*, *gpsB*, and *ponA* or mutations leading to specific amino acid substitutions or single domain deletions in FlhG and Hag. Corresponding complemented strains were generated by introducing wild‐type copies of either *gpsB* or *flhG* into the *amyE* locus, using plasmid pDR111. This plasmid allows expression of the complemented genes under the control of the IPTG‐dependent Phyper‐spank promoter (kind gift from David Rudner).

pDR111 was also used to construct a *B. subtilis* strain encoding a GpsB‐mScarlet fusion. The DNA fragment for the generation of the *gpsB*‐*mScarlet* construct was amplified with primers, which introduced the ribosome binding site of *B. subtilis* EF‐TU upstream of *gpsB* and a linker sequence (GSGLSGLGGGGGSL) between GpsB and mScarlet. The resulting PCR product was ligated into the SalI and SphI sites of pDR111. The resulting plasmid pAD306 was transformed into strain PB11, yielding strain ad08. The mNeonGreen fusions of FlhG and FliM were created using vector pSG1164 via a single‐crossover event at the native gene locus. pSG1164 harbors an xylose‐inducible promoter and produces a C‐terminal fusion of the target protein with mNeonGreen joined by a linker sequence (GSGLSGLGGGGGSL) (Strach et al. 2023). To generate the mNeonGreen‐fusion proteins, 500 bp of the 3′‐end of the target gene (excluding the stop‐codon) was amplified and integrated into the plasmid by ligation into the BamHI and EcoRI sites. The resulting plasmids (FliM, pAD323; FlhG, pAD321) were transformed into strain PB11 and ad08, which in addition also express the GpsB‐mScarlet fusion from the *amyE* locus.

### 2.2 Fluorescence Microscopy and Staining of Hook and Flagellin Proteins

For microscopy, *B. subtilis* strains were cultured in S750 medium, inoculated with an LB overnight culture to a starting OD_600_ of 0.05, and then incubated at 37°C until reaching an OD_600_ of 0.4–0.6. Antibiotics were supplemented as necessary: spectinomycin at 100 μg mL^−1^ and chloramphenicol at 5 μg mL^−1^. Induction of the GpsB‐mScarlet construct and complementation was done by adding 0.5 mM IPTG final concentration, while FlhG‐mNeonGreen and FliM‐mNeonGreen were induced using a final concentration of 0.5% (w/v) xylose.

Staining of the hook protein FlgE and the flagellin protein Hag was performed as previously described (Courtney et al. 2012). Briefly, after harvesting 1 mL of cell culture (6200*g* for 2 min), cells were washed with 1 mL of PBS (137 mM NaCl, 2.7 mM KCl, 10 mM Na_2_HPO_4_, and 2 mM KH_2_PO_4_, pH 8.0). The resulting pellet was resuspended in 50 μL of PBS. Fluorescence staining was done by the addition of 5 μg/mL^−1^ of Alexa Fluor 488 C_5_ Maleimide or Alexa Fluor 594 C_5_ Maleimide (Thermo Fisher Scientific), followed by an incubation for 10 min at room temperature. Subsequently, the cells were washed twice with 1 mL of PBS and resuspended in 100 μL of S750 medium. For microscopy, 5 μL of cells were spotted onto glass coverslips and overlaid with a 1% agarose pad (w/v) prepared with S750 medium.

SIM (Structured Illumination Microscopy) images were captured using a Zeiss ELYRA PS.1 microscope equipped with an ANDOR iXon 987 EMCCD camera and an alpha Plan‐Apochromat 100x/1.46 Oil DIC M27 Elyra objective, with exposure times of 50 ms and a gain setting of 5. Samples were excited at 488 nm (HR Diode 488–200, at 3% output) for imaging of mNeonGreen‐fusions or Alexa Fluor 488 C_5_ Maleimide‐stained proteins. mScarlet‐fusions or Alexa Fluor 594 C_5_ Maleimide stained proteins were excited at 561 nm (HR DPSS 561–200, 5% output). Detection of fluorescence signals was done with the following filter settings: BP 495–575/LP750 (green fluorescence) and BP 570–650/LP750 (red fluorescence). Z‐stacks of fifteen images (step size = 0.1 μm) were collected, with 3 rotations and 5 phases per plane. SIM reconstructions were processed using ZEN software by Zeiss. Image data analysis was performed using ImageJ. Three independent cultivations were analyzed for each strain. In each of these cultivations, signals from a minimum of 30 representative cells were manually quantified, resulting in approximately 100 cells analyzed per experiment.

### 2.3 Yeast Two‐Hybrid Analysis

For Y2H interaction assays, plasmids expressing the bait proteins, fused to the Gal4 DNA‐binding domain, and prey proteins, fused to the Gal4 activation domain, were co‐transformed into the reporter strain PJ69‐4A (James et al. 1996). Y2H interactions were documented by spotting representative transformants in 10‐fold serial dilution steps onto SC‐Leu‐Trp (−LT) and SC‐His‐Leu‐Trp (‐HLT; HIS3 reporter) plates, which were incubated for 3 days at 30°C. Growth on HLT plates is indicative of a moderate interaction.

### 2.4 Protein Production and Purification

*Escherichia coli* strain BL21 (DE3) (Novagen) transformed with pET24d‐ or pGAT3 constructs was used for protein production, with cells cultured in lysogeny broth medium (LB) supplemented with 1.5% (w/v) d(+)‐lactose monohydrate for 16 h at 30°C. Cells were harvested via centrifugation (3500*g*, 15 min, 4°C), and the resulting pellets were resuspended in appropriate buffers depending on the choice of the purification procedure: His lysis buffer (20 mM HEPES pH 8.0, 250 mM NaCl, 20 mM KCl, 20 mM MgCl_2_, 40 mM imidazole) for His‐tagged proteins, and SEC buffer (20 mM HEPES pH 7.5, 200 mM NaCl, 20 mM KCl, 20 mM MgCl_2_) for GST‐tagged proteins. Cells were lysed using a microfluidizer (M110‐L, Microfluidics), followed by centrifugation (48,000*g*, 20 min, 4°C) to remove cell debris. For protein purification, the cleared lysates were applied to either a HisTrapFF affinity column (Cytiva) or a GE Healthcare GSTrapFF column, depending on the protein tag. After loading, a wash step with 5 column volumes of the respective lysis buffer was performed. Subsequently, His‐tagged proteins were eluted with lysis buffer containing 250 mM imidazole, while GST‐tagged proteins were eluted using GSH buffer (SEC buffer with 50 mM Tris–HCl, 20 mM glutathione). Following elution, protein samples were concentrated using Amicon concentrators (Millipore) according to their molecular weight and underwent further purification by size exclusion chromatography (SEC) using an S200 XK16/600 column (GE Healthcare) equilibrated with SEC buffer. The fractions containing the desired proteins were pooled and concentrated once more for downstream applications.

### 2.5 GST‐Pulldown Assays

GST pulldown assays were carried out in SEC buffer (20 mM HEPES pH 7.5, 200 mM NaCl, 20 mM KCl, 20 mM MgCl_2_) using MoBiTec spin columns and filters. Each spin column was loaded with 30 μL of Protino Glutathione Agarose Beads (Macherey‐Nagel) and washed with 500 μL of SEC buffer at 1500 g for 1 min. Following this, 1 nmol of GST‐tagged protein was immobilized on the beads and rotated for 15 min, after which the beads were washed again. Next, putative interaction partners (10 nmol of protein) and, if required, nucleotides (2 nmol) were mixed with the GST‐protein loaded beads and incubated for 30 min while rotating. Afterward, the beads underwent three wash cycles, followed by an elution step with 40 μL of GSH buffer (SEC buffer with 50 mM Tris–HCl, 20 mM glutathione). The eluted samples were analyzed via SDS‐PAGE and Coomassie staining for protein interaction. The entire assay was carried out at room temperature.

### 2.6 ATPase Activity Assay

*Gt*FlhG and *Gt*FlhG‐ΔMTS (both at a concentration of 20 μM) were supplemented with 2 mM ATP and then incubated with or without *Gt*GpsB in SEC buffer (20 mM HEPES pH 7.5, 200 mM NaCl, 20 mM KCl, 20 mM MgCl_2_) at 37°C. Following a 1‐h incubation, the reaction was halted by adding chloroform, followed by vortexing for 15 s, boiling for 15 s at 98°C, and promptly flash‐freezing in liquid nitrogen for storage. For high‐performance liquid chromatography (HPLC) measurements, samples were thawed and subjected to centrifugation (17,300*g*, 15 min, 4°C), and the resulting aqueous phase containing the nucleotides was carefully transferred into new vials. Extracted nucleotides were analyzed using an Agilent 1260 Series HPLC system (Agilent Technologies) equipped with a Metrosep A Supp5–150/4.0 column (Metrohm International). Nucleotide measurements were carried out at 260 nm with a flow rate of 0.6 mL min^−1^ over 15 min in a buffer containing 90 mM (NH_4_)_2_CO_3_ at pH 9.25. All measurements were repeated with proteins from two independent purifications.

### 2.7 Western Blot Analysis

Western blot analysis was performed to assess protein stability and quantity. Samples (10 μL) from pulldown experiments or cell lysates of the respective strains (harvested from exponentially growing cultures and adjusted to an OD_600_ of 10) were loaded onto an SDS‐PAGE gel. Proteins were transferred using the TransTurbo Blot system (Bio‐Rad). For detection, an HRP‐conjugated 6 × His tag antibody was used for purified proteins, while specific primary antibodies against GpsB, Hag, and TufA were used, followed by corresponding HRP‐conjugated secondary antibodies. Chemiluminescence was employed for signal detection.

### 2.8 Transmission Electron Microscopy

Carbon‐coated copper grids (400 mesh) were hydrophilized by glow discharging (PELCO easiGlow, Ted Pella, USA). 5 μL of bacterial suspensions from the log phase were applied onto the hydrophilized grids and stained with 2% uranyl acetate after a short washing step with double‐distilled H_2_O. Samples were analyzed with a JEOL JEM‐2100 transmission electron microscope using an acceleration voltage of 120 kV. A 2 k F214 FastScan CCD camera (TVIPS, Gauting) was used for image acquisition.

## 3 Results

### 3.1 GpsB Interacts With FlhG and Phenocopies a *flhG* Deletion Strain

**FIGURE 1:**
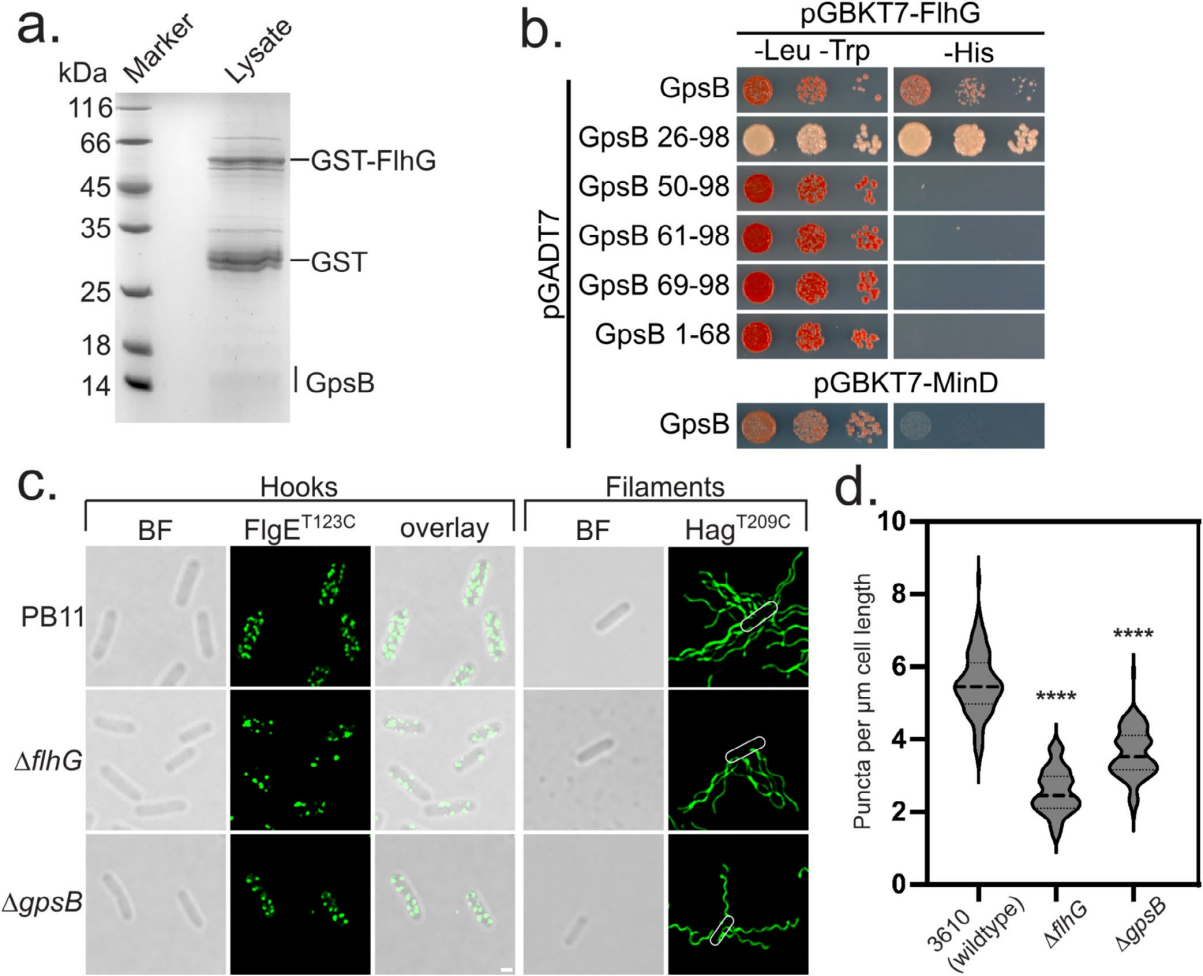
GpsB interacts with FlhG and impacts the spatial distribution of flagella in *B. subtilis* 3610. (a) Coomassie‐stained SDS‐PAGE of a lysate pulldown employing purified GST‐tagged *Bs*FlhG and a cytosolic extract from exponentially growing *B. subtilis* cells. (b) *Bs*FlhG, but not its close homologue *Bs*MinD (Ref. (Schuhmacher, Rossmann, Dempwolff, et al. 2015)), interacts with *Bs*GpsB in the Yeast two‐Hybrid (Y2H) assay. Selection on *‐Trp ‐Leu* ensures plasmid maintenance, while *‐His* selects for protein interaction by reporter gene activation. (c) Overlay of transmitted light and fluorescence micrographs showing *B. subtilis* wild type (3610), *flhG* and *gpsB* deletion cells carrying a Thr_123_Cys variant of the flagellar hook protein FlgE or a Thr209Cys mutation in the filament protein Hag, stained with Alexa Fluor 488 C_5_ Maleimide. (d) Violine plots showing the distribution of fluorescence hook signals per μm cell length from the same set of *B. subtilis* strains. 100 cells per strain were analyzed. Asterisks indicate significance level of *p* < 0.0001.

To elucidate FlhG's specific role in regulating flagella localization, we conducted Glutathione‐S‐transferase (GST) pulldown assays using *Bacillus subtilis* 3610 cell lysates. Initially, we generated GST‐tagged FlhG in *Escherichia coli* BL21(DE3) and purified the protein through a two‐step protocol, involving Ni‐ion affinity chromatography followed by size exclusion chromatography. The resulting GST‐FlhG was then incubated with lysates from *B. subtilis* 3610 in the logarithmic growth phase. Subsequently, we performed a GST pulldown assay and used mass spectrometry to identify potential interaction partners (Table S1). As a negative control, *B. subtilis* 3610 lysate was subjected to the GST pulldown assay without any added GST‐tagged protein, ensuring that observed interactions were specific to GST‐FlhG. Our pulldown experiments not only confirmed the known interaction with the FliY protein (Schuhmacher, Rossmann, Dempwolff, et al. 2015), but also suggested an interaction with the protein GpsB (Table S1) (Figure 1a).

To corroborate this discovery, we proceeded with a yeast two‐hybrid (Y2H) assay. Importantly, GpsB exhibited interaction with FlhG in the Y2H, but not with MinD, a closely related structural homologue of FlhG (Figure 1b). To gain further support for this conclusion, we created a *gpsB* knockout strain within *B. subtilis* 3610 and examined the distribution and quantity of its flagella. In order to visualize these flagella, we utilized a strain engineered with a threonine‐to‐cysteine substitution at position 123 in the hook protein FlgE and at position 209 of the flagellin protein Hag, facilitating the fluorescence‐staining of mature hooks and flagella using Alexa Fluor 488 C_5_ Maleimide (Courtney et al. 2012). Microscopy of the *gpsB* deletion strain revealed a flagellation pattern distinct from the wild type and partially reminiscent of the *flhG* deletion strain, characterized by a significantly irregular flagellation pattern and a reduced number of focal points in both the *gpsB* and *flhG* mutant (Figure 1c; Figure S1). Whereas the wild type exhibited an average of 5.5 +/−0.9 puncta per μm of cell length, both the *gpsB* and *flhG* deletion strains displayed reduced counts, with 3.6 +/−0.7 and 2.6 +/−0.6 puncta, respectively (Figure 1d). Complementation of the *gpsB* and *flhG* deletion strains with *gpsB* and *flhG*, respectively, restored the wild‐type situation (Figure S2). To further investigate hook distribution on the cell surface, we utilized transmission electron microscopy with negative staining. Our analysis revealed that the *gpsB* and *flhG* mutants exhibited large regions devoid of hooks compared to the wild type. Additionally, the *flhG* mutant displayed prominent clustering of hook regions (Figure S3). Finally, Western blot analysis of Hag protein identified a reduced level of flagellin in the *gpsB* and *flhG* mutants (Figure S4). Thus, our findings strongly suggest a link between the spatial–temporal organization of flagella and GpsB, adding a further function to GpsB, which was initially identified as a regulator of cell wall synthesis during growth in *B. subtilis* (Tavares et al. 2008; Claessen et al. 2008; Pompeo et al. 2015).

### 3.2 GpsB Interacts With the FlhG Homodimer Without Affecting the FlhG ATPase Activity

**FIGURE 2:**
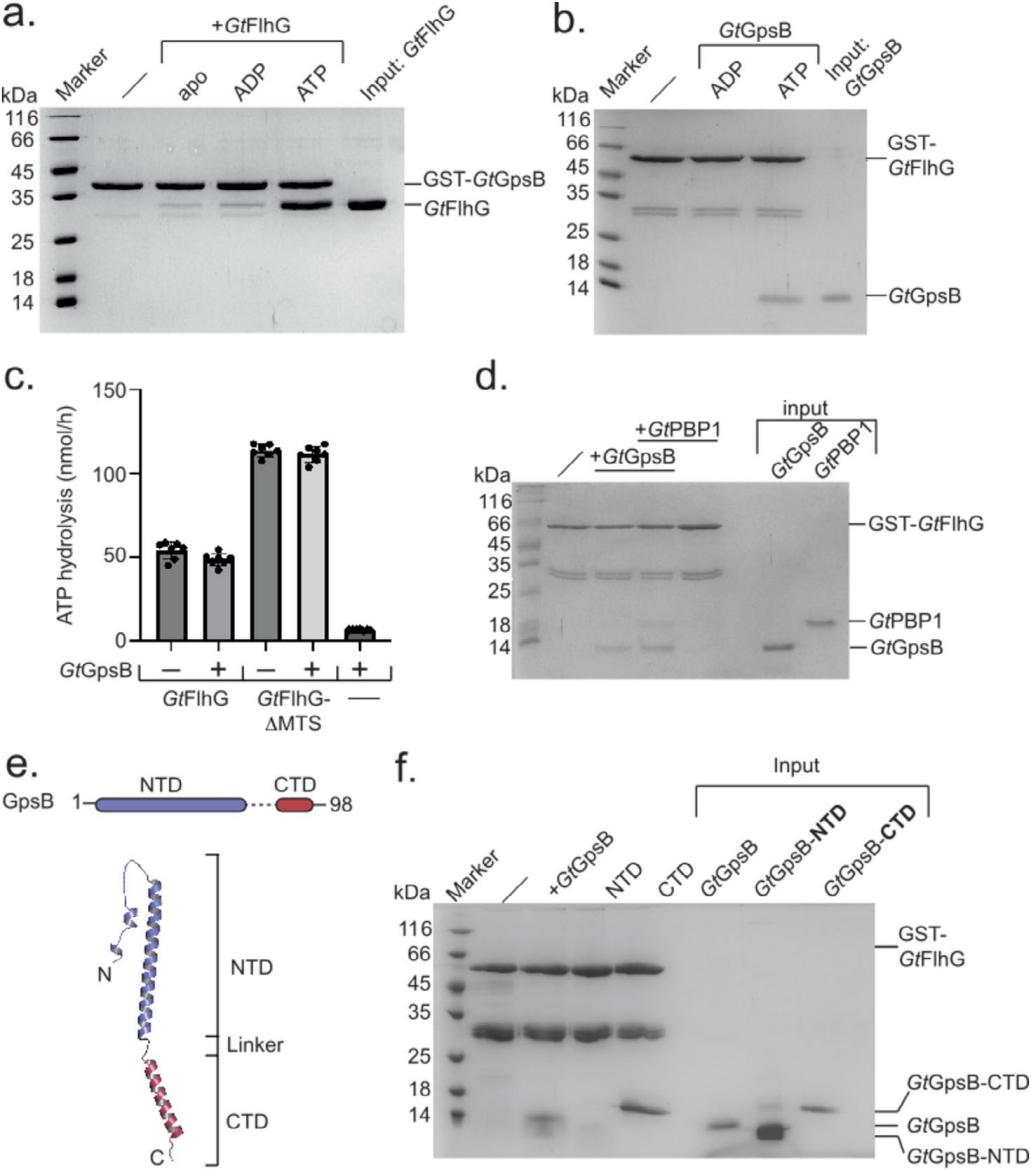
The FlhG‐GpsB interaction. (a) Coomassie‐stained SDS‐PAGE of an in vitro pulldown assay investigating the interaction of a GST‐tagged *Gt*GpsB with *Gt*FlhG in the presence of no nucleotide, 2 mM ADP, and ATP. (b) The same experiment as in a., now using GST‐tagged *Gt*FlhG as bait and *Gt*GpsB as prey in the presence of either 2 mM ADP or ATP. (c) ATP hydrolysis assay investigating whether *Gt*GpsB would impact the ATPase activity of *Gt*FlhG or its MTS‐lacking variant. All measurements were performed in multiple replicates (shown as dots). Columns show the mean value and standard deviation of replicates. Values of individual measurements are shown as points. (d) Coomassie‐stained SDS‐PAGE of an in vitro pulldown assay employing GST‐tagged *Gt*GpsB as bait and either *Gt*PBP1^1‐33^ alone or *Gt*PBP1^1‐33^ and *Gt*FlhG as prey. The experiments were performed in the presence of 2 mM ATP. (e) *B. subtilis* GpsB. *Upper panel*: Domain structure of *Bs*GpsB with its N‐ and C‐terminal domains in blue and red, respectively. *Lower panel*: Alpha Fold model of a *Bs*GpsB monomer. N‐ and C‐termini are indicated by ‘N' and ‘C', respectively. (f) Coomassie‐stained SDS‐PAGE employing a GST‐tagged *Gt*FlhG as bait and different variants of *Gt*GpsB as prey. NTD and CTD versions of *Gt*GpsB were purified as His‐tagged and His‐GB1‐fusions, respectively. The experiment was performed in the presence of 2 mM ATP.

To further probe the interaction between FlhG and GpsB, we chose to utilize the closely related orthologs obtained from the moderate thermophile *Geobacillus thermodenitrificans* NG80‐2 (*Gt*) (Figure S5). *Gt*GpsB and *Gt*FlhG, derived from this organism, displayed superior structural stability compared to their counterparts from the mesophilic *B. subtilis* . Initially, we conducted an in vitro pulldown assay using a GST‐tagged *Gt*GpsB as the bait and *Gt*FlhG as the prey to confirm the interaction of the *G. thermodenitrificans* NG80‐2 proteins (Figure S6). When incubated individually, *Gt*GpsB and *Gt*FlhG exhibited only a sub‐stoichiometric interaction (Figure 2a; Apo). Given that FlhG is capable of ATP‐dependent homodimerization (12, 13), we subsequently investigated the impact of the nucleotides ADP and ATP on the *Gt*GpsB‐*Gt*FlhG interaction. While no change in interaction strength was noted for ADP (Figure 2a; ADP), in the presence of ATP, the *Gt*FlhG‐*Gt*GpsB interaction became stoichiometric (Figure 2a; ATP). In order to exclude the possibility that the *Gt*GpsB or *Gt*FlhG shows unspecific binding to the GST‐beads, we conducted a reverse experiment. This time, we utilized a GST‐tagged *Gt*FlhG as the bait and *Gt*GpsB as the prey (Figure 2b). As observed for GST‐tagged *Gt*GpsB, a stoichiometric interaction between *Gt*FlhG and *Gt*GpsB was discerned only in the presence of ATP. A *Gt*FlhG mutant deficient in ATP hydrolysis (D60A) retained GpsB binding activity, while a variant deficient in ATP binding (K36A) lost the ability to interact with GpsB (Figure S7).

The requirement for ATP in the *Gt*FlhG‐*Gt*GpsB interaction suggests a potential role for *Gt*GpsB in stimulating the ATPase activity of *Gt*FlhG. To explore this hypothesis, we conducted HPLC‐based ATPase assays using either *Gt*FlhG or *Gt*GpsB, or a combination of both proteins at a 1 to 2 ratio of *Gt*FlhG to *Gt*GpsB. These experiments did not reveal an impact of *Gt*GpsB on the ATPase activity of *Gt*FlhG. Interaction of the C‐terminal membrane targeting sequence (MTS) of *Gt*FlhG with the cell membrane was shown to increase basal *Gt*FlhG ATPase activity (Schuhmacher, Rossmann, Dempwolff, et al. 2015). The interaction of *Gt*FlhG with the membrane results in the release of the MTS from its binding pocket on the *Gt*FlhG surface. This membrane‐binding dependent increase in basal ATPase activity can be mimicked in vitro with a construct lacking the C‐terminal MTS. Of note, *Gt*FlhG‐ΔMTS interacted with *Gt*GpsB, identical to wild‐type (Figure S8). As shown for the full‐length *Gt*FlhG construct, no increase in ATPase activity was observed for *Gt*FlhG‐ΔMTS upon *Gt*GpsB interaction (Figure 2c). Taken together, these experiments provide evidence that the interaction between *Gt*GpsB and *Gt*FlhG depends upon the presence of ATP, and concurrently, *Gt*GpsB exerts no influence on the ATPase activity of *Gt*FlhG, neither of the full‐length protein nor in a construct mimicking the membrane‐bound state of *Gt*FlhG. Furthermore, the stringent dependence on ATP for a meaningful *Gt*FlhG‐*Gt*GpsB interaction strongly suggests that *Gt*GpsB engages specifically with the ATP‐dependent homodimeric state of *Gt*FlhG.

### 3.3 GpsB Can Simultaneously Interact With FlhG and PBP1

A key function of GpsB in Gram‐positive bacteria is to engage with the central peptidoglycan synthase, PBP1 (penicillin‐binding protein 1, encoded by *ponA*) (21–23). Therefore, we investigate whether *Gt*GpsB could engage in simultaneous interactions with both *Gt*PBP1 and *Gt*FlhG or if the binding of these proteins to *Gt*GpsB would be mutually exclusive. Consequently, we conducted in vitro pulldown assays utilizing GST‐tagged *Gt*GpsB as bait and the 33 N‐terminal amino acids of *Gt*PBP1 and *Gt*FlhG as potential prey. The results revealed that *Gt*PBP1 interacted with *Gt*GpsB, confirming previous observations. Furthermore, *Gt*FlhG demonstrated its ability to interact with *Gt*GpsB even in the presence of *Gt*PBP1, suggesting that both proteins can concurrently engage in interactions with *Gt*GpsB (Figure 2d). A *ponA* mutant showed no obvious perturbation of the flagellation pattern, suggesting PBP1 does not contribute to the decision of flagella positioning (Figure S9).

In earlier structural analyses of GpsB, it was revealed that the protein comprises an N‐terminal domain (NTD) and a C‐terminal domain (CTD), interconnected by a flexible linker (Figure 2e). The NTD forms homodimers, whereas the CTD exhibits a robust capability to form dimers or trimers. Notably, the NTD of GpsB seems to function as an interaction platform for penicillin‐binding proteins (PBPs), including PBP1 (21–23). Therefore, we investigated whether a GST‐tagged *Gt*FlhG would interact with the NTD or the CTD of *Gt*GpsB. Our experiment demonstrated that both full‐length *Gt*GpsB and its CTD could interact with *Gt*FlhG, whereas the NTD of *Gt*GpsB did not exhibit this interaction (Figure 2f). Thus, we conclude that *Gt*FlhG interacts with the CTD of *Gt*GpsB. This observation agrees well with the finding that *Gt*PBP1 and *Gt*FlhG can simultaneously interact with *Gt*GpsB through their N‐ and C‐terminal domains, respectively.

### 3.4 FlhG Can Simultaneously Bind GpsB and the Flagellar C‐Ring

**FIGURE 3:**
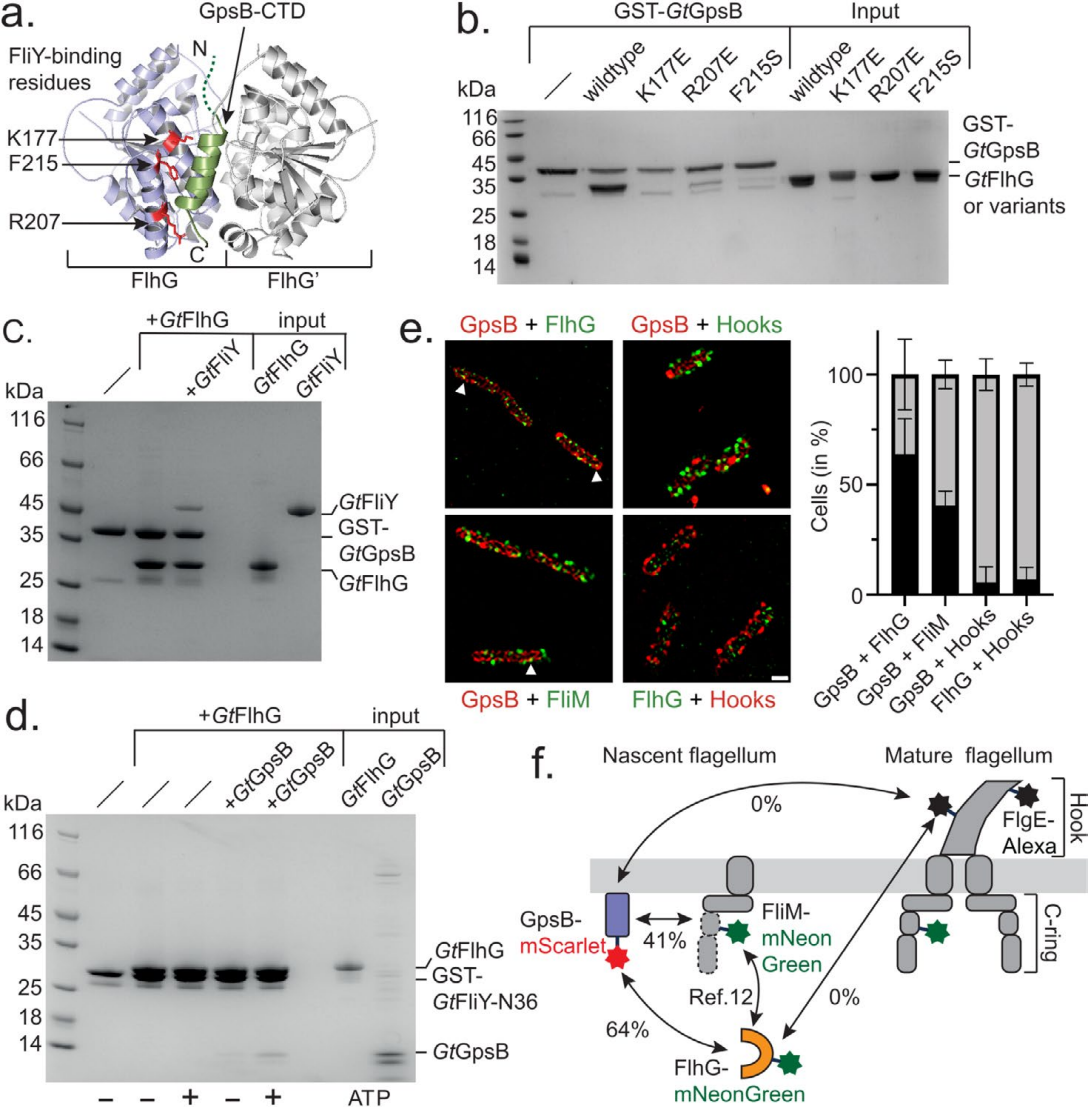
FlhG provides a binding platform for the cell cycle regulator GpsB and the C‐ring protein FliY. (a) AlphaFold model of the FlhG homodimer bound to the C‐terminal domain (CTD) of GpsB. Residues that were previously identified to be crucial for the binding of FliY (Ref: (Schuhmacher, Rossmann, Dempwolff, et al. 2015)) are also shown. (b) Coomassie‐stained SDS‐PAGE of an in vitro pulldown assay employing GST‐tagged *Gt*GpsB as bait and different *Gt*FlhG variants previously shown to be defective in FliY‐binding. (c) In vitro pulldown assay employing GST‐tagged *Gt*GpsB as bait and either *Gt*FlhG alone or *Gt*FlhG and FliY as prey. (d) In vitro pulldown assay employing GST‐tagged FliY‐N36 as bait and either *Gt*FlhG alone or *Gt*FlhG and *Gt*GpsB as prey. The impact of ATP was also studied and is indicated with a ‘+’. (e) Colocalization experiments. *Left side*: Representative images of co‐localization experiments of GpsB (red) with either FlhG (green, *upper left*), hooks (green, *upper right*), and FliM (green, *lower left*). In the lower right panel, co‐staining of FlhG (green) and hooks (red) is shown. Arrows indicate co‐localization events. *Right side*: Quantification of cells (in percent) showing at least one co‐localization event of the given protein pairs. The errors represent the standard deviation of 100 cells in three independent biological replicates. (f) Graphical summary of the experiments and their outcomes is shown in Figure 3e. The percent values represent the frequency of cells in which protein colocalization was observed.

In a recent study, we demonstrated the interaction between FlhG and the flagellar C‐ring protein FliY, pinpointing residues Lysine 177, Arginine 277, and Phenylalanine 215 on FlhG as crucial for this interaction (Schuhmacher, Rossmann, Dempwolff, et al. 2015). To predict a potential binding site of *Gt*GpsB on *Gt*FlhG, we made use of Alphafold2 (Senior et al. 2020). In this prediction, *Gt*GpsB interacted with a *Gt*FlhG region, which was very similar to the binding site of FliY, irrespective of whether the interaction was modeled with a monomer or a trimer of the *Gt*GpsB‐CTD (Figure 3a). Modeling using the *B. subtilis* proteins predicted an identical interaction for GpsB and FlhG (Figure S10). Thus, our *in silico* analysis suggested the possibility of overlapping binding sites for GpsB and FliY on FlhG.

To evaluate this prediction, we conducted in vitro pulldown assays employing a GST‐tagged *Gt*GpsB as bait and variants of *Gt*FlhG containing mutations in the FliY binding surface at Lysine 177, Arginine 277, and Phenylalanine 215 as prey (Figure 3a). These experiments reveal that altering the residues on *Gt*FlhG known to interact with *Gt*FliY also impacts its ability to interact with *Gt*GpsB (Figure 3b). However, while substitutions at these residues completely abolish the interaction between FlhG and FliY, the R207E and F215S mutations still allow a weak interaction between FlhG and GpsB. This finding supports our hypothesis that GpsB and FliY share partially overlapping binding sites on FlhG.

Considering that an ATP‐dependent FlhG homodimer offers two binding sites suitable for either FliY or GpsB, we aimed to investigate whether the binding of FliY and GpsB is mutually exclusive or if binding to both proteins could occur simultaneously. To distinguish between both possibilities, we used a GST‐tagged *Gt*GpsB and examined the interaction with either *Gt*FlhG, *Gt*FliY, or both proteins in the presence of ATP, a crucial prerequisite for *Gt*GpsB to bind with the *Gt*FlhG dimer. These experiments suggest that both proteins can concurrently bind to an ATP‐bound *Gt*FlhG dimer (Figure 3c). To further support this observation, we tested the binding of *Gt*GpsB and *Gt*FliY to *Gt*FlhG in a competition experiment. Increasing the amount of *Gt*FliY did not affect the ability of *Gt*GpsB to simultaneously bind *Gt*FlhG, reinforcing that the interactions of both proteins with *Gt*FlhG are not mutually exclusive (Figure S11). Previously, we demonstrated that the interaction between FliY and FlhG requires solely the N‐terminal 36 amino acid residues of FliY (i.e., FliY‐N36), which are both necessary and sufficient (12). Therefore, we also performed an additional experiment now immobilizing GST‐*Gt*FliY‐N36, and studied its interaction with *Gt*FlhG and *Gt*GpsB. As demonstrated earlier (12), the interaction between *Gt*FliY‐N36 and *Gt*FlhG was robust and occurred independently of ATP (Figure 3d). When *Gt*GpsB was introduced, an interaction between the *Gt*FliY‐N36/*Gt*FlhG complex was only discernible in the presence of ATP (Figure 3d). This observation further supported the idea that the ATP‐dependent homodimer of *Gt*FlhG is capable of concurrently interacting with both *Gt*GpsB and *Gt*FliY.

Having demonstrated that the ATP‐dependent *Gt*FlhG homodimer can simultaneously bind to *Gt*GpsB and the C‐ring protein *Gt*FliY in vitro, we aimed to further corroborate these findings within the cellular context. To this end, we conducted in vivo co‐localization studies in *B. subtilis* . Initially, we utilized IPTG‐inducible mScarlet‐labeled *Bs*GpsB produced from the *amyE* site of the *B. subtilis* chromosome and mNeonGreen‐labeled *Bs*FlhG produced from the native *flhG* site under control of its native promoter in the wild‐type background (Figure 3e; Figure S12). We observed one or more overlapping signals of both protein variants in approximately 63 +/−13% of all cells analyzed (Figure 3e,f). Subsequently, we investigated the colocalization of *Bs*GpsB with mNeonGreen‐labeled *Bs*FliM, a direct interaction partner of the *Bs*FliY marker of the C‐ring (Schuhmacher, Rossmann, Dempwolff, et al. 2015). In this setup as well, we found that both proteins co‐localized in approximately 40.6 +/−5% of all cells analyzed (Figure 3e,f). Anticipating that *Bs*GpsB would primarily associate with nascent flagellar C‐rings, we further examined the spatial distribution of GpsB in relation to flagellar hooks, a definitive indicator of mature C‐rings (Kubori et al. 1992; Minamino and Macnab 1999; Erhardt and Hughes 2010). These experiments revealed that hook and *Bs*GpsB signals were mutually exclusive (Figure 3e,f). The same was true for FlhG and hooks (Figure 3e,f), underscoring the concerted action of FlhG and GpsB at nascent and not mature flagella. In the FliM‐mNeonGreen versus hook control, almost perfect colocalization was observed in 100% of the cells (Figure S12).

### 3.5 FlhG‐GpsB Locally Restrains Stimulation of the SRP‐GTPase FlhF

**FIGURE 4:**
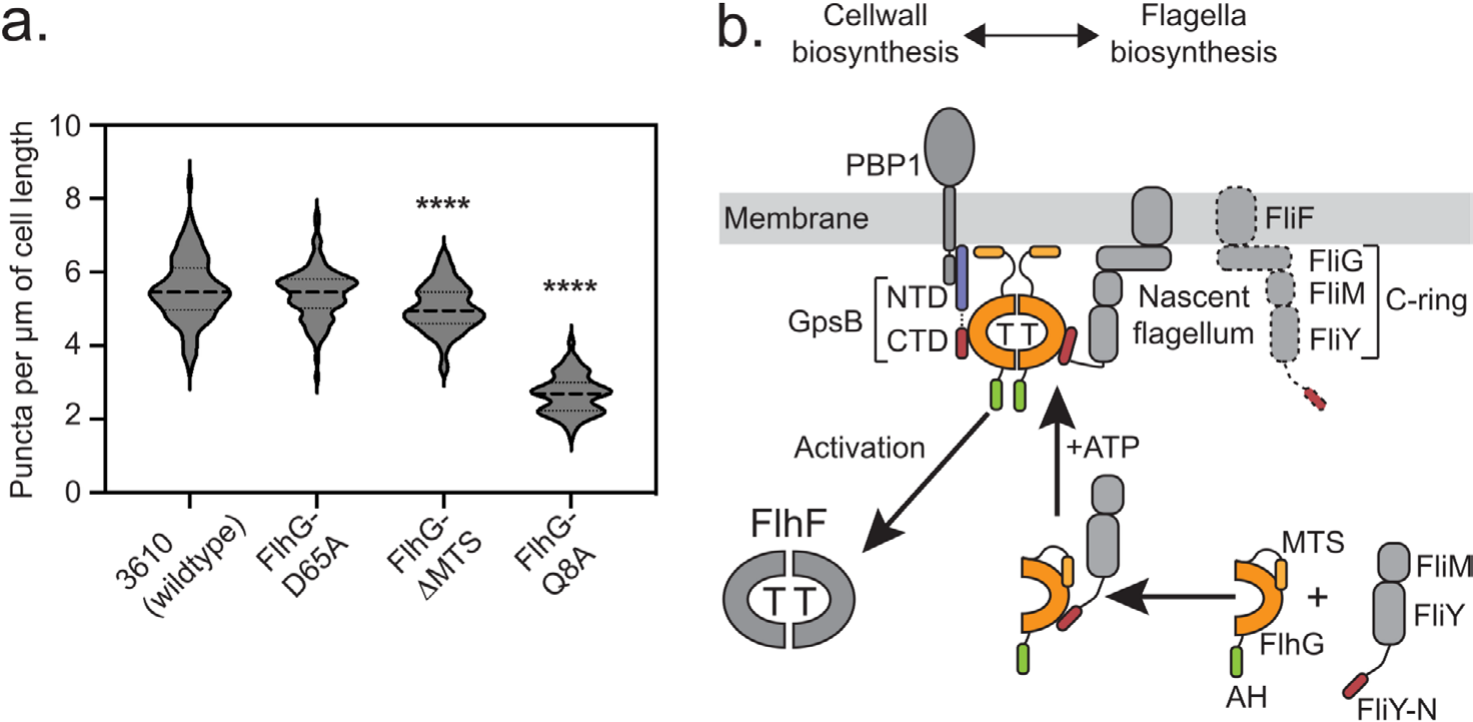
FlhG‐GpsB exerts local restraint on the activation of the SRP‐GTPase FlhF. (a) Impact of individual mutations in *flhG* on the number of puncta derived from hook staining per μm of cell length. Violine plots showing the distribution of fluorescence hook signals per μm cell length from the same set of *B. subtilis* strains. 100 cells per strain were analyzed. Asterisks indicate significance level of *p* < 0.0001. (b) The model elucidates how FlhG (orange and green for ATP‐MTS and activator helix (AH), respectively) collaborates with the cell wall regulator GpsB (blue and red for its N‐ and C‐terminal domains, respectively) and the C‐ring proteins FliM and FliY (both gray with FliY‐N in red) to facilitate the localization of peritrichous flagella in *B. subtilis* .

Finally, we wanted to understand to what extent the ATPase activity of FlhG, which is not affected by GpsB (see above), contributes to the distribution of flagella. To visualize flagella, we again employed a *B. subtilis* strain engineered with a threonine‐to‐cysteine substitution at position 123 in the hook protein FlgE (see above, (Courtney et al. 2012)). Initially, we investigated the *Bs*FlhG variant D65A, which is incapable of ATP hydrolysis, leading to the formation of a constitutive homodimer (Schuhmacher, Rossmann, Dempwolff, et al. 2015). This variant showed no discernible effect on the distribution of flagellar hooks (Figure 4a), suggesting that ATPase activity is not essential for the spatial localization of flagella. Subsequently, we examined a *Bs*FlhG variant lacking its membrane targeting sequence, which displayed only a modest but significant impact with approximately 10% fewer puncta per μm of cell length (Figure 4a). If neither ATPase activity nor its interaction with the membrane is are critical factor, what then is the primary function of FlhG?

It has been shown that glutamine 8 within the conserved ‘DQAxxLR’ motif in the N‐terminus of FlhG is critical to stimulate the GTPase activity of the SRP‐GTPase FlhF (Bange et al. 2011; Rossmann et al. 2015). Introduction of an alanine substitution at Q8 in *Bs*FlhG resulted in a significant alteration in puncta per cell (Figure 4a), closely resembling the deletion phenotypes observed for *flhG*. Consequently, we infer that the stimulation of FlhF GTPase activity represents the central function of FlhG in ensuring the accurate distribution of flagella.

## 4 Discussion

The maintenance of a specific number and arrangement of flagella, a pivotal early taxonomic criterion in microbiology, is essential for bacterial movement. Despite this significance, the mechanisms responsible for consistently establishing these patterns in each cell division cycle remain poorly understood.

The regulatory module formed by the proteins FlhG and FlhF plays a crucial role in the spatial‐numerical control of flagella across bacterial species, excluding γ‐proteobacteria. FlhF ensures proper flagella localization, while FlhG acts as a numerical regulator, typically limiting flagellar numbers to one (reviewed in: (Schuhmacher, Thormann, et al. 2015; Kazmierczak and Hendrixson 2013)). Consequently, the deletion of *flhG* results in the formation of multiple polar flagella. However, current perspectives on the mechanisms of the control of flagellation pattern are mostly influenced by studies on polar and amphitrichous flagellates. In the Gram‐positive model bacterium *B. subtilis* , which features peritrichous flagellation, each cell displays around 26 regularly arranged flagella with a minimum separation of approximately 0.4 μm (Guttenplan et al. 2013). In lieu of controlling flagellar numbers, FlhG appears to influence the nearest neighbor distance of flagella. Its absence leads to flagellar aggregation or “clumped” flagella (Guttenplan et al. 2013, *this study*). These findings prompt the question of how FlhG precisely governs the nearest neighbor distance of flagella. In *B. subtilis* , FlhG displays a highly dynamic behavior, signifying its cyclic movement between the cytoplasm and the basal bodies/membrane (Schuhmacher, Rossmann, Dempwolff, et al. 2015). This dynamic behavior stems from FlhG's feature to transition between an Apo/ADP‐bound monomer and an ATP‐bound homodimer (Schuhmacher, Rossmann, Dempwolff, et al. 2015; Chanchal and Banerjee 2017; Bange and Sinning 2013). Specifically, FlhG is capable of interacting with negatively charged lipids only in its homodimer state, rendering it competent for membrane interactions (Schuhmacher, Rossmann, Dempwolff, et al. 2015).

Independent of nucleotides, FlhG interacts with the very N‐terminus of the flagellar C‐ring protein FliY, which together with FliM constitutes the “lower” flagellar C‐ring of the flagellar basal body (Figure 4b). Thus, FlhG captures the FliY/FliM complex in the cytoplasm and participates in the assembly of the complex to FliG at nascent flagellar structures (Schuhmacher, Rossmann, Dempwolff, et al. 2015; Blagotinsek et al. 2020). Moreover, in vitro investigations have demonstrated FlhG‐dependent assembly of oligomeric FliM/FliY structures at FliG in the presence of lipids and ATP, essential for FlhG homodimerization (Schuhmacher, Rossmann, Dempwolff, et al. 2015). This observation implies that the homodimer plays a role in orchestrating the assembly of FliM/FliY/FliG. Nevertheless, the function of the ATP‐dependent homodimer remained enigmatic. In the present study, we have identified the cell cycle regulator GpsB to interact with the ATP‐bound homodimer state of FlhG. Recently, GpsB has also been shown to act as an adaptor protein, which connects a major class A peptidoglycan synthase (PBP1) with other cell wall and cell cycle proteins, and to cell shape determinants (Cleverley et al. 2016; Cleverley et al. 2019; Rismondo et al. 2016). The observation that GpsB can simultaneously interact with PBP1 and FlhG suggests that peptidoglycan remodeling may also play a role in flagellar positioning and assembly. GpsB shares an overlapping binding site with FliY at FlhG. GpsB is part of the late *B. subtilis* cell division machinery (Halbedel and Lewis 2019; Tavares et al. 2008). In *B. subtilis* , GpsB re‐localized from the lateral sides of the cell cylinder in young, shorter growing cells to the division septum as soon as longer, fully grown cells started to divide (Claessen et al. 2008). Thus, our study shows that FlhG interferes with the cell division/elongation machinery of *B. subtilis* in order to enable the correct distance between the laterally arranged flagella. In summary, our study unveils an elegant mechanism that intricately links cell wall synthesis and the formation of emerging flagella. This mechanism serves to synchronize the consistent growth and elongation of cells with the orderly development of membrane‐cell wall‐spanning macromolecular machinery, exemplified by the flagellum.

## Author Contributions

**Anita Dornes:** writing – original draft, investigation, formal analysis. **Patrica Bedrunka:** investigation. **Dieter Kressler:** methodology, investigation. **Thomas Heimerl:** methodology. **Jan Pané‐Farré:** supervision, writing – original draft, conceptualization, investigation. **Gert Bange:** conceptualization, funding acquisition, writing – original draft, validation, supervision.

## Conflicts of Interest

The authors declare no conflicts of interest.

## Supporting information


Table S1.

**Figure S1.** Filament stains. Overlay of transmitted light and fluorescence micrographs showing cells of *B. subtilis* wild type (3610), *flhG* and *gpsB* deletion mutants, and their complementation strains carrying a Thr_209_Cys mutation in the filament protein Hag, stained with Alexa Fluor 488 C_5_ Maleimide.
**Figure S2.** Complementation experiments. The *gpsB*, *flhG*, and *flhG* mutants are shown on the left side (‘Experiment’) and the corresponding strains complemented with wildtype copies of either *gpsB* or *flhG* on the right side (‘Complementation’). Wild‐type copies are integrated at the *amyE* locus, regulated by an IPTG‐inducible P_hyp_ promoter. Violine plots showing the distribution of fluorescence hook signals per μm cell length from the complementation strains. 100 cells per strain were analyzed. Asterisks denote significance levels, with **** indicating *p* < 0.0001 and *** indicating *p* < 0.001, for differences of approximately < 10% in puncta numbers between mutant and wild‐type cells.
**Figure S3.** *Transmission electron microscopy*. Representative micrographs of *B. subtilis* wild‐type, *flhG*, and *gpsB* mutant strains grown to mid‐log phase and analyzed by transmission electron microscopy. Flagellar hooks are indicated by star symbols. Scale bars represent 1 μm.
**Figure S4.** Western blot analysis of Hag protein. Cultures of *B. subtilis* wild‐type, *flhG*, and *gpsB* mutants were grown to mid‐log phase, adjusted to an OD_600_ of 10, lysed, and analyzed by Western blot using a specific antibody against the filament protein Hag. The elongation factor Tu was detected as a loading control to ensure equal cell amounts.
**Figure S5.** *Sequence similarity analysis of GpsB across bacterial species*. The GpsB sequences from *Geobacillus thermodenitrificans* (*Gt*), *Bacillus subtilis* (*Bs*), *Listeria monocytogenes* (*Lm*), *Streptococcus pneumoniae* (*Sp*), and *Staphylococcus aureus* (*Sa*) were analyzed for sequence similarity using NCBI BLAST. Similarity was assessed based on E‐value, sequence identities, positives, and gaps. Sequence alignment was performed using the BLOSUM62 scoring matrix.
**Figure S6.** Interaction between FlhG and GpsB is conserved between *B. subtilis* and *Geobacillus thermodenitrificans* NG80. Coomassie‐stained SDS‐PAGE showing in vitro pulldown assay employing GST‐tagged FlhG as bait and GpsB as prey for the *B. subtilis* (*Bs*) and *G. thermodenitrificans* (*Gt*) proteins on the left and right, respectively.
**Figure S7.** Influence of ATP binding and hydrolysis on the interaction between GpsB and FlhG. Coomassie‐stained SDS‐PAGE analysis of an in vitro pulldown assay using GST‐tagged *Gt*GpsB as bait. The interaction was tested with wild‐type *Gt*FlhG, a D60A mutant (which can bind but not hydrolyze ATP), and a K36A mutant (which cannot bind ATP). The effect of ATP addition was also examined and is indicated by a ‘+’ symbol. Samples were incubated at room temperature for an hour before SDS‐PAGE loading to evaluate the impact of ATP hydrolysis in the wild‐type and catalytically dead FlhG variant.
**Figure S8.** The MTS of FlhG does not impact the ATP‐dependent FlhG‐GpsB interaction. Coomassie‐stained SDS‐PAGE of an in vitro pulldown assay investigating the interaction of a GST‐tagged *Gt*GpsB with *Gt*FlhG lacking its MTS (*Gt*FlhG‐ΔMTS) in the presence of no nucleotide, 2 mM ADP, and ATP.
**Figure S9.** Hook stain of a *ponA* mutant. Overlay of transmitted light and fluorescence micrographs showing *B. subtilis* wild type (3610) and a *ponA* deletion carrying a Thr_123_Cys variant of the flagellar hook protein FlgE, stained with Alexa Fluor 488 C_5_ Maleimide. Violine plots showing the distribution of fluorescence hook signals per μm cell length for both strains. 100 cells per strain were analyzed.
**Figure S10.** Alphafold prediction of *B. subtilis* FlhG and the GpsB‐CTD and sequence comparison between *B. subtilis* and *G. thermodenitrificans* of FlhG. Residues previously identified in FlhG as crucial for FliY binding are highlighted in red and marked in the sequence alignment by asterisks. Sequence alignment was performed using the BLOSUM62 scoring matrix.
**Figure S11.** Competition pulldowns. (a) In vitro pulldown assay employing a GST‐tagged *Gt*GpsB as bait and *Gt*FliY as prey. (b) SDS‐PAGE of an in vitro pulldown assay using GST‐*Gt*FlhG as the bait and *Gt*GpsB and *Gt*FliY as the prey. *Gt*FliY was added in increasing amounts (1, 5, and 10 nmol) to the reactions. A Western blot of this assay was performed using a 6x‐His tag‐specific antibody. The experiments were performed in the presence of 2 mM ATP.
**Figure S12.** Colocalization experiments. Overlay of transmitted light and fluorescence micrographs of co‐localization experiments of GpsB (red) with either FlhG (green), hooks (green), or FliM (green). Additionally, FlhG (green) with hooks (red) and FliM (green) with hooks (red) are shown as controls. Arrows indicate co‐localization events. Violine plots showing distribution of fluorescence hook signals per μm cell length from the same set of *B. subtilis* strains. 100 cells per strain were analyzed. Asterisks indicate significance level of *p* < 0.0001.
**Table S2.** Strains used in this study.
**Table S3.** Plasmids used in this study.
**Table S4.** Primers used in this study.

## Data Availability

The data that support the findings of this study are available from the corresponding author upon reasonable request.
