# Supplementary material for: FlhG Cooperates With the Cell Cycle Regulator GpsB to Confine Peritrichous Flagella in B. subtilis
Source: Mol Microbiol. 2025 May 19;124(2):131–40. doi: 10.1111/mmi.15375 (PMC12327844; doi:10.1111/mmi.15375)
Supplement: Supplementary file 2 — Figure S1. Filament stains. Overlay of transmitted light and fluorescence micrographs showing cells of B. subtilis wild type (3610), flhG and gpsB deletion mutants, and their complementation strains carrying a Thr209Cys mutation in the filament protein Hag, stained with Alexa Fluor 488 C5 Maleimide. Figure S2. Complementation experiments. The gpsB, flhG, and flhG mutants are shown on the left side (‘Experiment’) and the corresponding strains complemented with wildtype copies of either gpsB or flhG on the right side (‘Complementation’). Wild‐type copies are integrated at the amyE locus, regulated by an IPTG‐inducible Phyp promoter. Violine plots showing the distribution of fluorescence hook signals per μm cell length from the complementation strains. 100 cells per strain were analyzed. Asterisks denote significance levels, with **** indicating p < 0.0001 and *** indicating p < 0.001, for differences of approximately < 10% in puncta numbers between mutant and wild‐type cells. Figure S3. Transmission electron microscopy. Representative micrographs of B. subtilis wild‐type, flhG, and gpsB mutant strains grown to mid‐log phase and analyzed by transmission electron microscopy. Flagellar hooks are indicated by star symbols. Scale bars represent 1 μm. Figure S4. Western blot analysis of Hag protein. Cultures of B. subtilis wild‐type, flhG, and gpsB mutants were grown to mid‐log phase, adjusted to an OD600 of 10, lysed, and analyzed by Western blot using a specific antibody against the filament protein Hag. The elongation factor Tu was detected as a loading control to ensure equal cell amounts. Figure S5. Sequence similarity analysis of GpsB across bacterial species. The GpsB sequences from Geobacillus thermodenitrificans (Gt), Bacillus subtilis (Bs), Listeria monocytogenes (Lm), Streptococcus pneumoniae (Sp), and Staphylococcus aureus (Sa) were analyzed for sequence similarity using NCBI BLAST. Similarity was assessed based on E‐value, sequence identities, positives, and ga [file MMI-124-131-s002.docx]

*Supplementary information for:*

**FlhG cooperates with the cell cycle regulator GpsB to confine peritrichous flagella in *B. subtilis*.**

Anita Dornes^1^, Patrica Bedrunka^1^, Dieter Kressler^2^, Thomas Heimerl^1^, Jan Pané-Farré^1,*^ and Gert Bange^1,2,3*^

^1^Philipps-University Marburg, Center for Synthetic Microbiology (SYNMIKRO) and Department of Chemistry, Karl-von-Frisch Strasse 14, 35043 Marburg, Germany

^2^Department of Biology University of Fribourg, Chemin du Musée 10, CH-1700 Fribourg, Switzerland

^3^Max Planck Institute for terrestrial Microbiology, Molecular Physiology of Microbes, Karl-von-Frisch Strasse 14, 35043 Marburg

*Correspondence: [gert.bange@synmikro.uni-marburg.de](mailto:gert.bange@synmikro.uni-marburg.de) or [panefarj@uni-marburg.de](mailto:panefarj@uni-marburg.de)

The file contains:

Supplementary Figures S1 – S12

Supplementary Tables S2 – S4


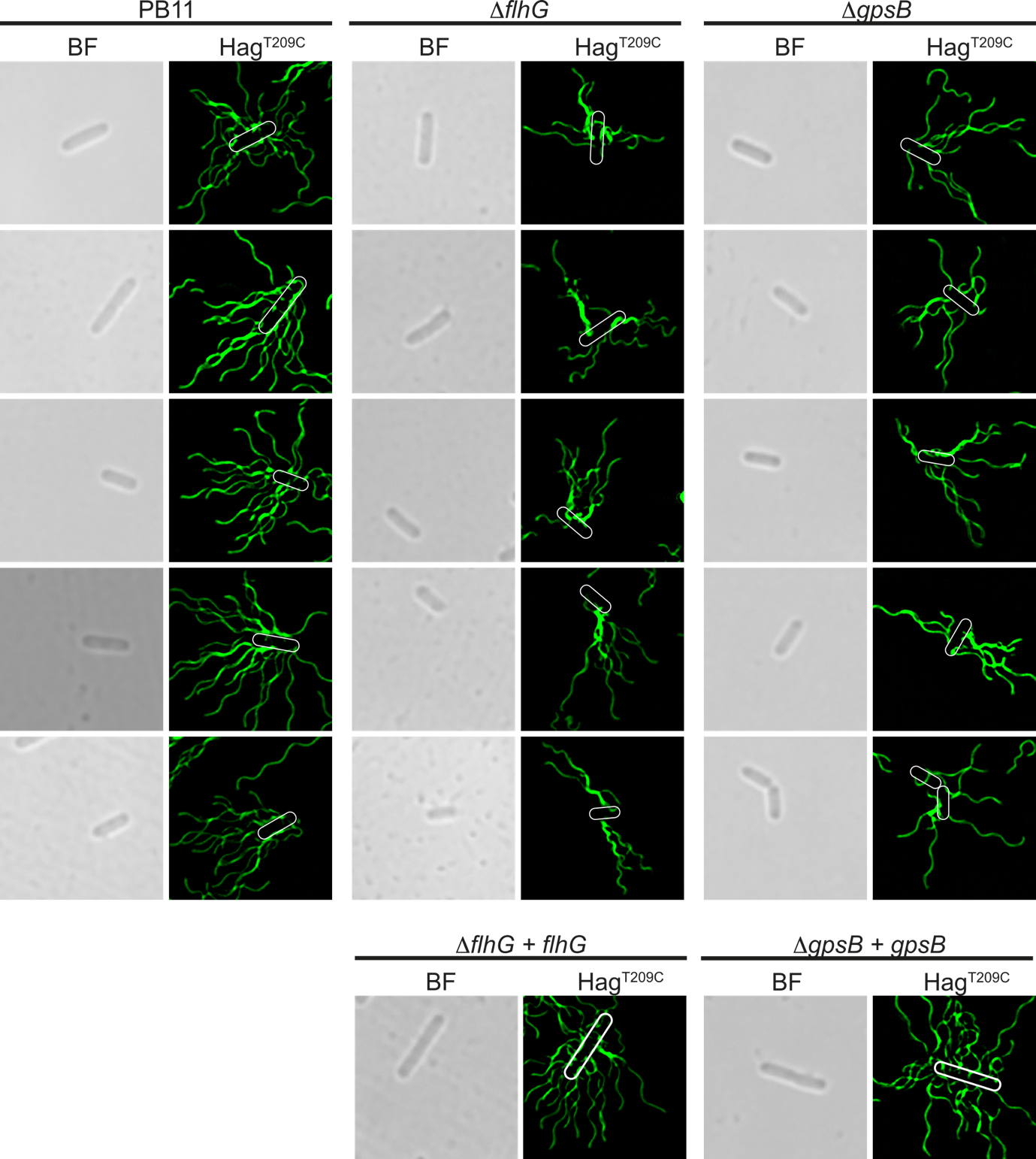


**Supplementary Fig. S1** Filament stains. Overlay of transmitted light and fluorescence micrographs showing cells of *B. subtilis* wild type (3610), *flhG* and *gpsB* deletion mutants and their complementation strains carrying a Thr_209_Cys mutation in the filament protein Hag stained with Alexa Fluor™ 488 C_5_ Maleimide.


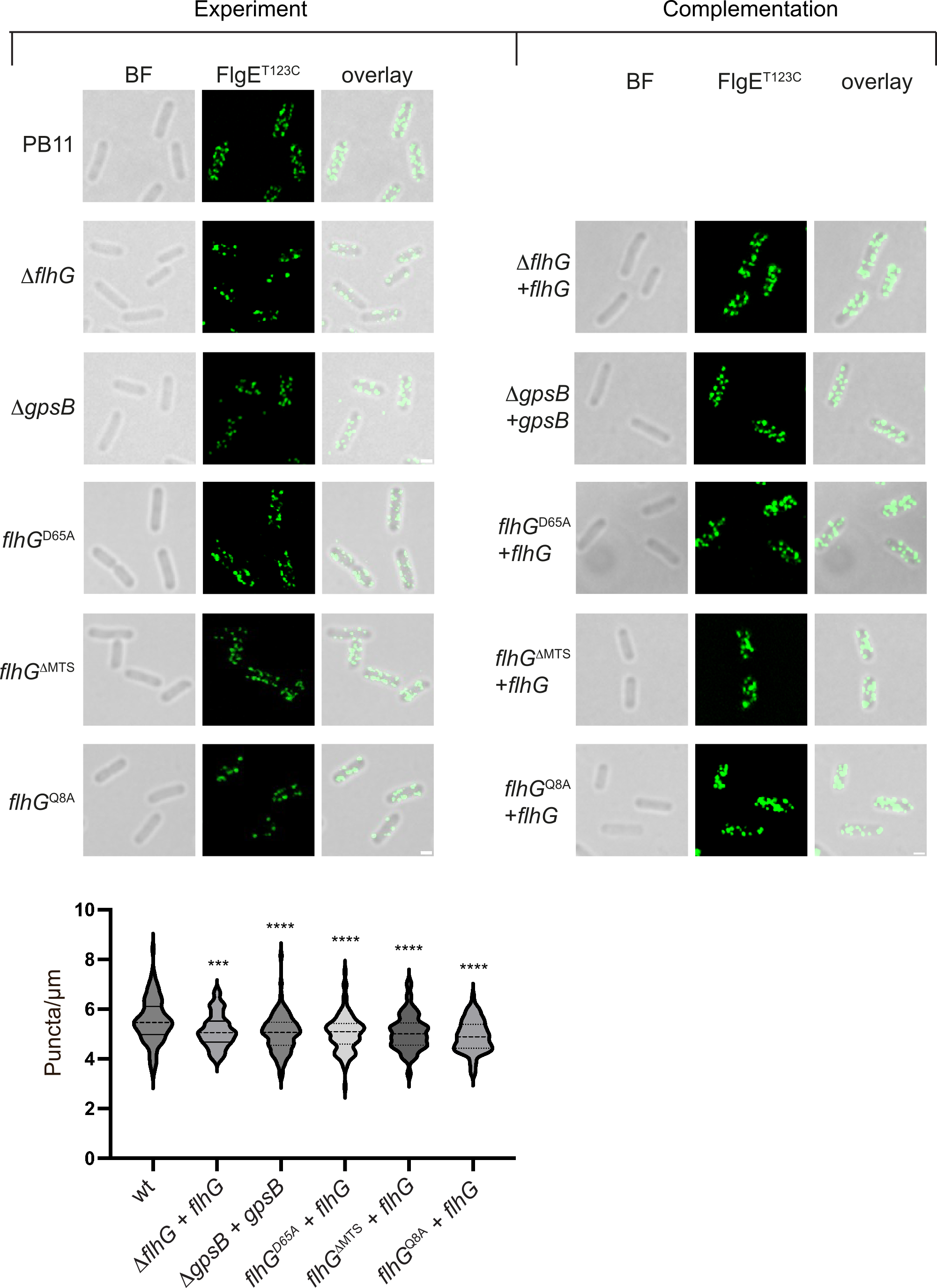


**Supplementary Fig. S2**. Complementation experiments. The *gpsB*, *flhG* and *flhG* mutants are shown on the left side (‘Experiment’) and the corresponding strains complemented with wildtype copies of either *gpsB* or *flhG* on the right side (‘Complementation’). Wild type copies are integrated at the *amyE* locus, regulated by an IPTG-inducible P_hyp_ promoter. Violine plots showing distribution of fluorescence hook signals per μm cell length from the complementation strains. 100 cells per strain were analyzed. Asterisks denote significance levels, with **** indicating p<0.0001 and *** indicating p<0.001, for differences of approximately <10% in puncta numbers between mutant and wild-type cells..


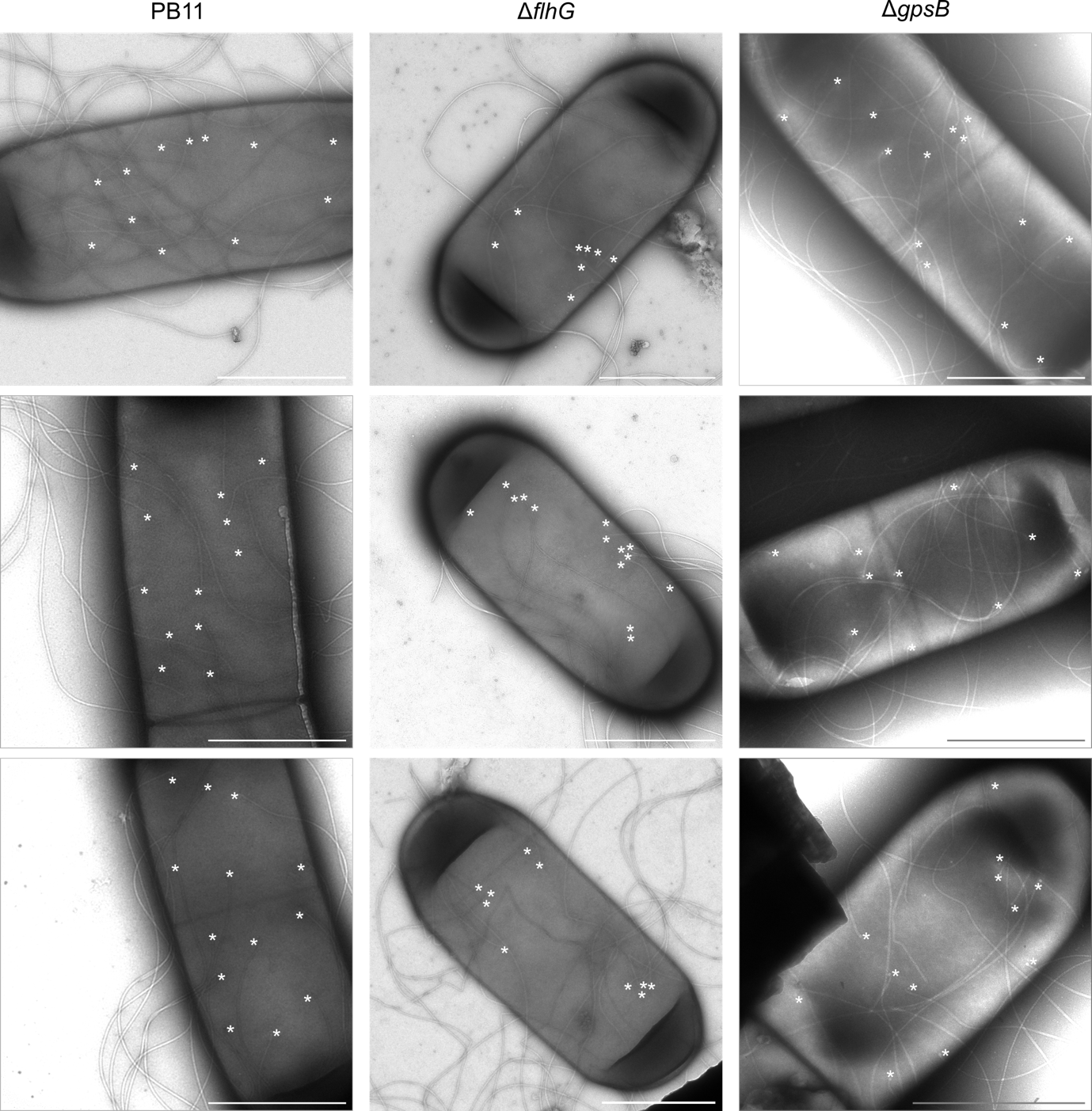


**Supplementary Fig. S3** Transmission electron microscopy. Representative micrographs of B. subtilis wild-type, flhG, and gpsB mutant strains grown to mid-log phase and analyzed by transmission electron microscopy. Flagellar hooks are indicated by star symbols. Scale bars represent 1 µm.


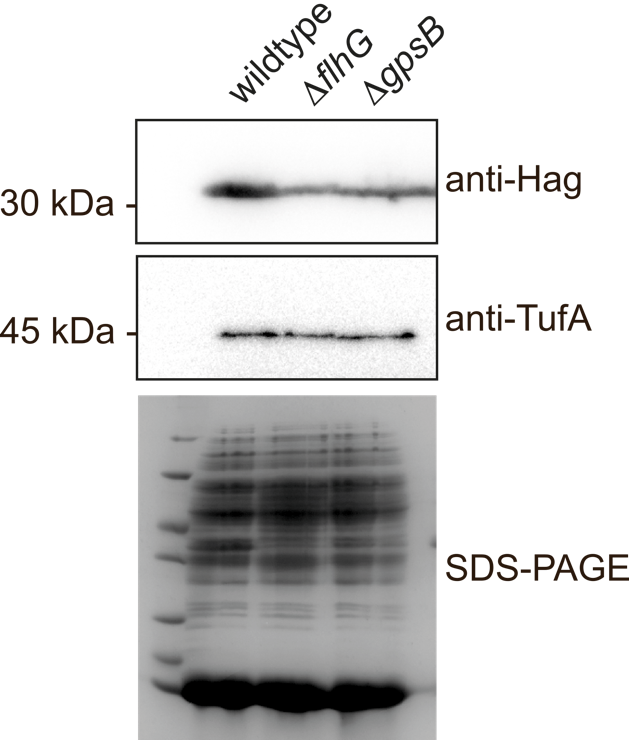


**Supplementary Fig. S4** Western blot analysis of Hag protein. Cultures of B. subtilis wild-type, flhG, and gpsB mutants were grown to mid-log phase, adjusted to an OD₆₀₀ of 10, lysed, and analyzed by Western blot using a specific antibody against the filament protein Hag. The elongation factor Tu was detected as a loading control to ensure equal cell amounts.


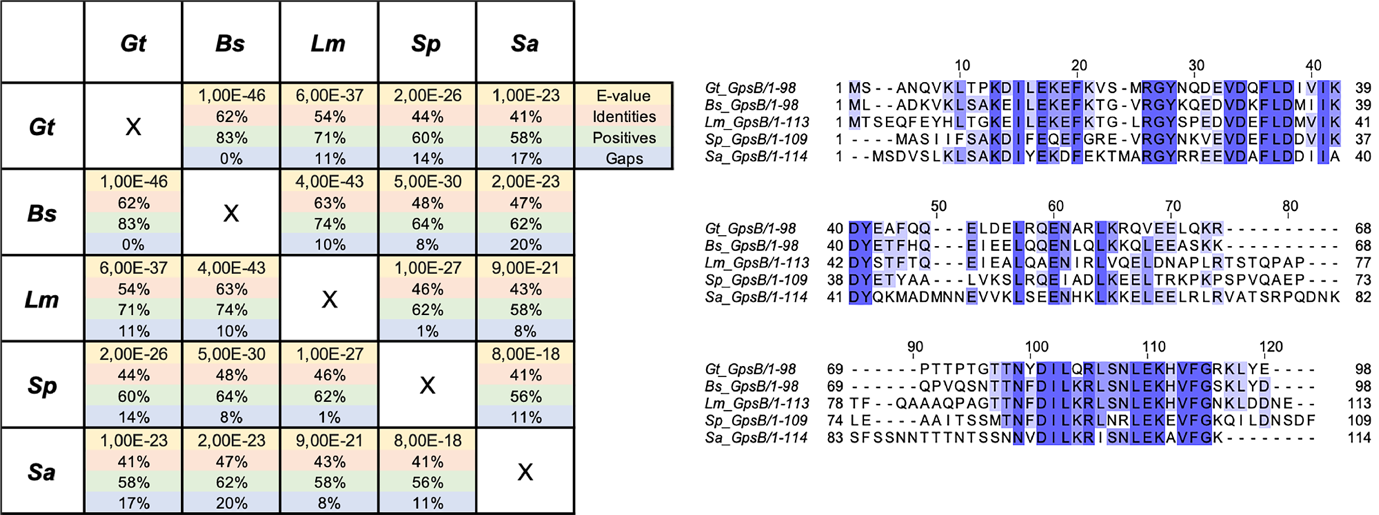


**Supplementary Fig. S5** Sequence similarity analysis of GpsB across bacterial species. The GpsB sequences from Geobacillus thermodenitrificans (*Gt*), Bacillus subtilis (*Bs*), Listeria monocytogenes (*Lm*), Streptococcus pneumoniae (*Sp*), and Staphylococcus aureus (*Sa*) were analyzed for sequence similarity using NCBI BLAST. Similarity was assessed based on E-value, sequence identities, positives, and gaps. Sequence alignment was performed using the BLOSUM62 scoring matrix.


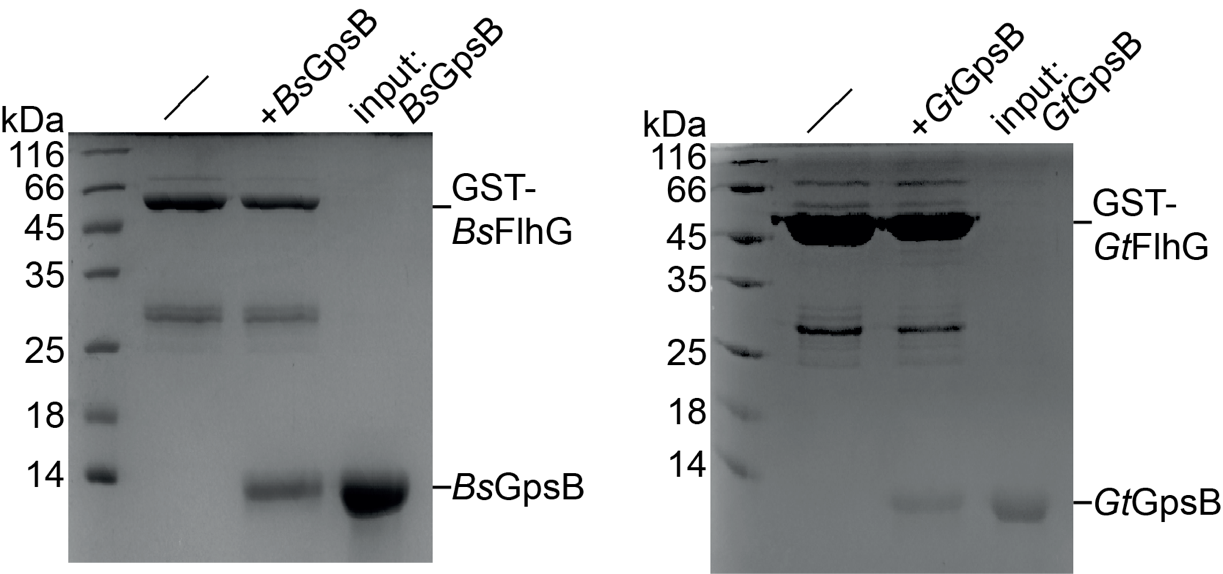


**Supplementary Fig. S6**. Interaction between FlhG and GpsB is conserved between *B. subtilis* and *Geobacillus thermodenitrifcans* NG80. Coomassie-stained SDS-PAGES showing in vitro pulldown assay employing GST-tagged FlhG as bait and GpsB as prey for the *B. subtilis* (*Bs*) and *G. thermodenitrificans* (*Gt*) proteins on the left and right, respectively.


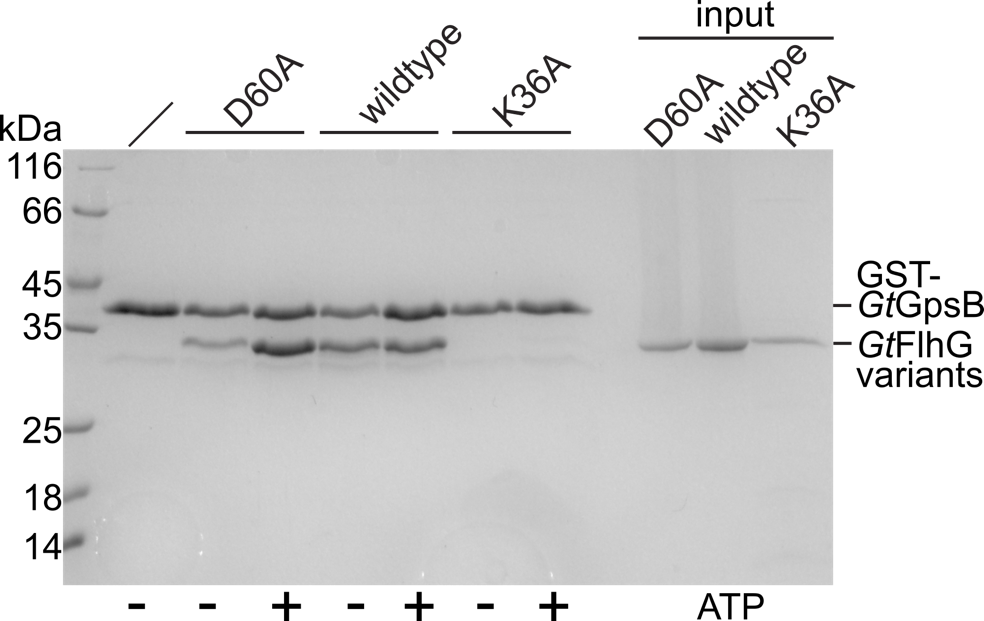


**Supplementary Fig. S7** Influence of ATP binding and hydrolysis on the interaction between GpsB and FlhG. Coomassie-stained SDS-PAGE analysis of an in vitro pulldown assay using GST-tagged *Gt*GpsB as bait. The interaction was tested with wild-type *Gt*FlhG, a D60A mutant (which can bind but not hydrolyze ATP), and a K36A mutant (which cannot bind ATP). The effect of ATP addition was also examined and is indicated by a ‘+’ symbol. Samples were incubated at room temperature for one hour before SDS-PAGE loading to evaluate the impact of ATP hydrolysis in the wild-type and catalytically dead FlhG variant.


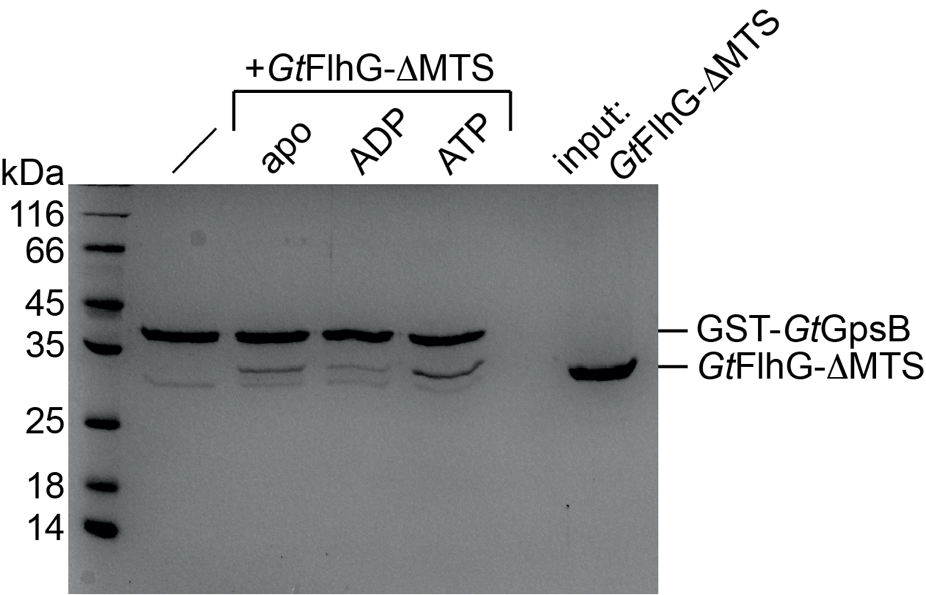


**Supplementary Fig. S8**. The MTS of FlhG does not impact the ATP-dependent FlhG-GpsB interaction. Coomassie-stained SDS-PAGE of an *in vitro* pulldown assay investigating the interaction of a GST-tagged *Gt*GpsB with *Gt*FlhG lacking its MTS (*Gt*FlhG-ΔMTS) in the presence of no nucleotide, 2 mM ADP and ATP.


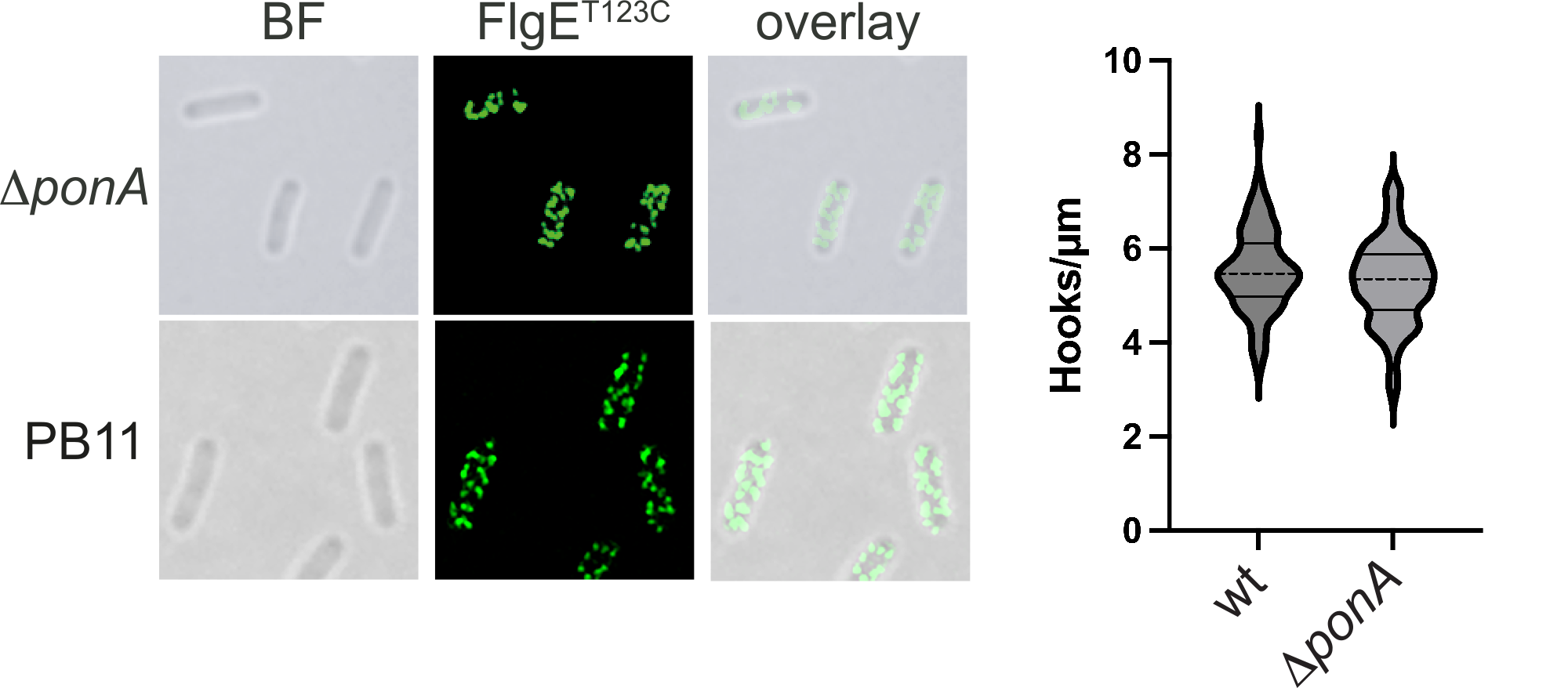


**Supplementary Fig. S9** Hook stain of a *ponA* mutant. Overlay of transmitted light and fluorescence micrographs showing *B. subtilis* wild type (3610) and a *ponA* deletion carrying a Thr_123_Cys variant of the flagellar hook protein FlgE stained with Alexa Fluor™ 488 C_5_ Maleimide. Violine plots showing distribution of fluorescence hook signals per μm cell length for both strains. 100 cells per strain were analyzed.


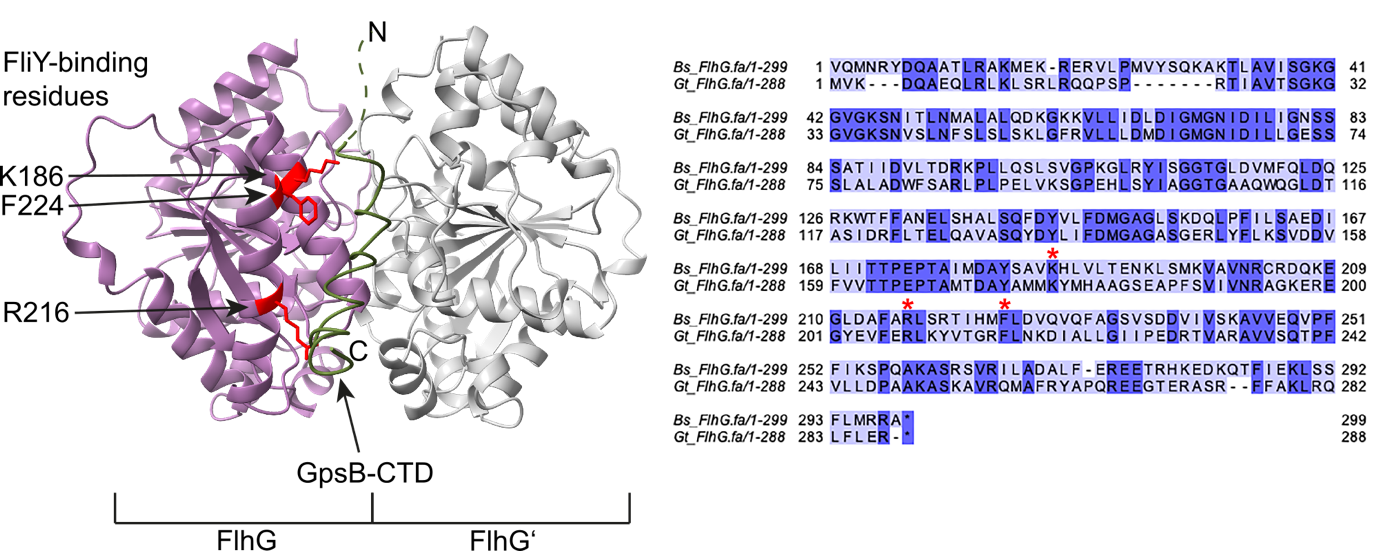


**Supplementary Fig. S10** Alphafold prediction of *B. subtilis* FlhG and the GpsB-CTD and sequence comparison between *B. subtilis* and *G. thermodenitrificans* of FlhG. Residues previously identified in FlhG as crucial for FliY binding are highlighted in red and marked in the sequence alignment by asterisks. Sequence alignment was performed using the BLOSUM62 scoring matrix.


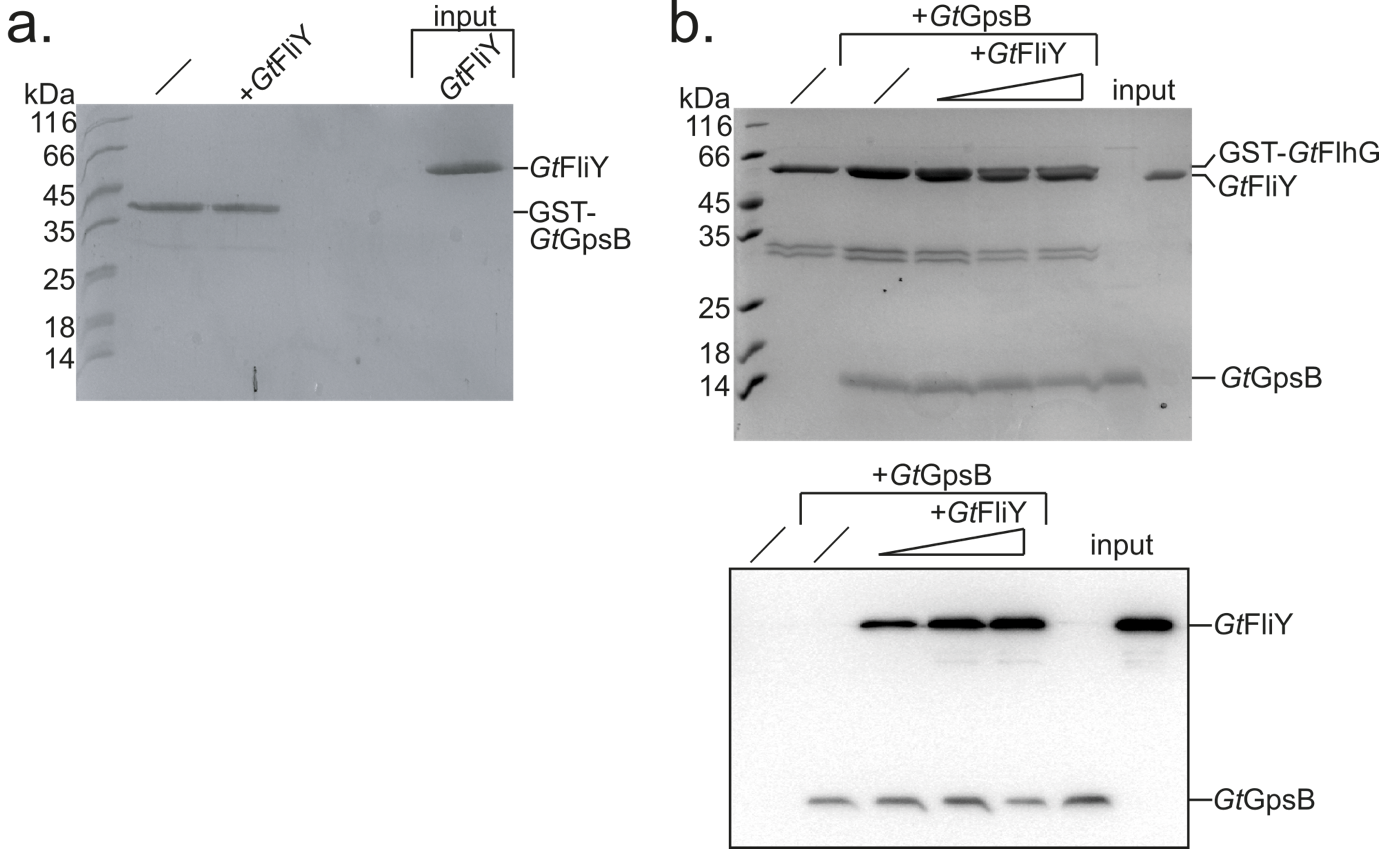


**Supplementary Fig. S11** Competition pulldowns. **a.** *In vitro* pulldown assay employing a GST-tagged *Gt*GpsB as bait and *Gt*FliY as prey. **b.** SDS-PAGE of an in vitro pulldown assay using GST-*Gt*FlhG as the bait and *Gt*GpsB and *Gt*FliY as the prey. *Gt*FliY was added in increasing amounts (1, 5 and 10 nmol) to the reactions. A Western blot of this assay was performed using a 6x-His tag specific antibody. The experiments were performed in the presence of 2 mM ATP.


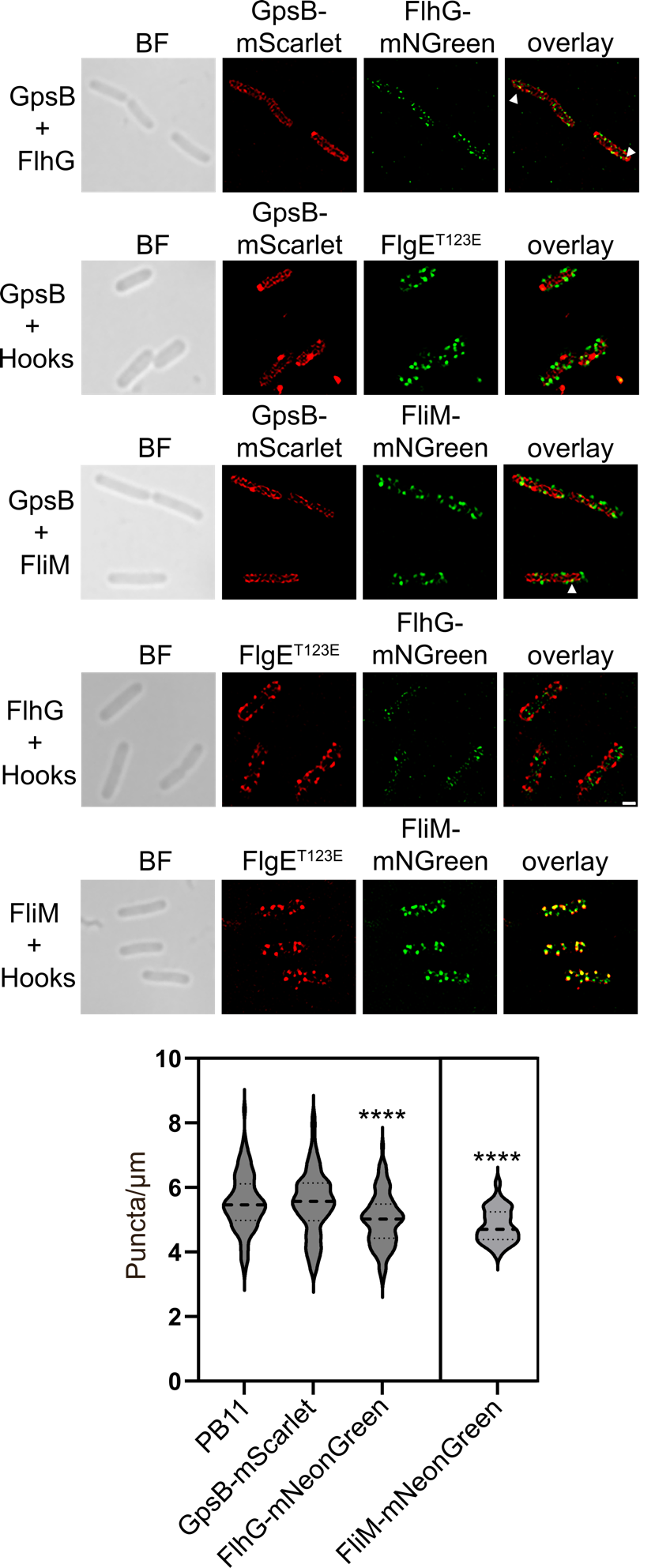


**Supplementary Fig. S12** Colocalization experiments. Overlay of transmitted light and fluorescence micrographs of co-localization experiments of GpsB (red) with either FlhG (green), hooks (green) or FliM (green). Additionally, FlhG (green) with hooks (red), and FliM (green) with hooks (red) are shown as controls. Arrows indicate co-localization events. Violine plots showing distribution of fluorescence hook signals per µm cell length from the same set of *B. subtilis* strains. 100 cells per strain were analyzed. Asterisks indicate significance level of p<0.0001.

**Supplementary Table S2. Strains used in this study.**

| Name | Genotype/ description | Source |
| --- | --- | --- |
| NCIB 3610 | wildtype | (1) |
| PB11 | *flgE*^T123C^ | *This study* |
| AD04 | Δ*flhG* *flgE*^T123C^ | *This study* |
| AD05 | Δ*gpsB* *flgE*^T123C^ | *This study* |
| AD06 | *flhG*^Q8A^ *flgE*^T123C^ | *This study* |
| AD07 | *flhG*^Δ279-296^ *flgE*^T123C^ | *This study* |
| AD08 | *amyE::P*_hyspank_*gpsP-mScarlet* *flgE*^T123C^ | *This study* |
| AD09 | *flhG-mNeonGreen flgE*^T123C^ | *This study* |
| AD10 | *flhG-mNeonGreen amyE::P*_hyspank_*gpsP-mScarlet* *flgE*^T123C^ | *This study* |
| AD11 | *fliM-mNeonGreen amyE::P*_hyspank_*gpsP-mScarlet* *flgE*^T123C^ | *This study* |
| AD12 | Δ*flhG* *flgE*^T123C^ *amyE::P*_hyspank_*flhG* | *This study* |
| AD13 | Δ*gpsB* *flgE*^T123C^ *amyE::P*_hyspank_*gpsB* | *This study* |
| AD14 | *flhG*^D65A^ *flgE*^T123C^ *amyE::P*_hyspank_*flhG* | *This study* |
| AD15 | *flhG*^Δ279-296^ *flgE*^T123C^ *amyE::P*_hyspank_*flhG* | *This study* |
| AD16 | *flhG*^Q8A^ *flgE*^T123C^ *amyE::P*_hyspank_*flhG* | *This study* |
| JPF07 | *flhG*^D65A^ *flgE*^T123C^ | *This study* |
| AD17 | *flgE*^T123C^ *hag*^T209C^ | *This study* |
| AD18 | Δ*flhG flgE*^T123C^ *hag*^T209C^ | *This study* |
| AD19 | ΔgpsB *flgE*^T123C^ *hag*^T209C^ | *This study* |
| AD20 | Δ*flhG flgE*^T123C^ *hag*^T209C^ *amyE::P*_hyspank_*flhG* | *This study* |
| AD21 | ΔgpsB *flgE*^T123C^ *hag*^T209C^ *amyE::P*_hyspank_gpsB | *This study* |
| AD22 | Δ*ponA flgE*^T123C^ | *This study* |

**Supplementary Table S3. Plasmids used in this study.**

| Name | Genotype/ description | Source |
| --- | --- | --- |
| pGBKT7 | For Y2H interaction assays: pGBKT7 plasmid expressing the bait protein fused to the Gal4 DNA-binding domain | Clontech |
| pGADT7 | For Y2H interaction assays: pGADT7 plasmid expressing the prey protein fused to Gal4 activation domain | Clontech |
| pET24d | Overproduction of His-tagged proteins | Novagen |
| pGAT3 | Overproduction of GST-tagged proteins | Novagen |
| pEMGB1 | Overproduction of His-GB1-tagged proteins | (2) |
| pMAD | Markerless deletion or mutation in *B. subtilis* | (3) |
| pSG1164 | Integration vector to fuse protein to mNeonGreen under control of a xylose-inducible promotor | (4) |
| pDR111 | Integration of genes into *amyE* under control of Phyper-spank promotor | Unpublished; kind gift of D. Rudner |
| pPB11 | Markerless Mutation of *flgE*^T123C^ | *This study* |
| pAD125 | Markerless deletion of *flhG* | *This study* |
| pAD126 | Markerless deletion of *gpsB* | *This study* |
| pAD161 | Heterologous overproduction of GST-*Bs*FlhG | *This study* |
| pAD193 | Heterologous overproduction of GST-*Gt*GpsB | *This study* |
| pAD185 | Heterologous overproduction of C-terminal His-tagged *Gt*FlhG | *This study* |
| pAD199 | Heterologous overproduction of GST-*Gt*FlhG | *This study* |
| pAD187 | Heterologous overproduction of N-terminal His-tagged *Gt*GpsB | *This study* |
| pAD218 | Heterologous overproduction of N-terminal His-tagged *Gt*FlhGΔMTS | *This study* |
| pAD220 | Heterologous overproduction of His-GB1-tagged *Gt*PBP1^1-33^ | *This study* |
| pAD200 | Heterologous overproduction of N-terminal His-tagged *Gt*GpsB-NTD (1-76) | *This study* |
| pAD201 | Heterologous overproduction of N-terminal His-tagged *Gt*GpsB-CTD (76-94) | *This study* |
| pJSS041 | Heterologous overproduction of C-terminal His-tagged *Gt*FlhG^K177E^ | (5) |
| pJSS045 | Heterologous overproduction of C-terminal His-tagged *Gt*FlhG^K207E^ | (5) |
| pJSS048 | Heterologous overproduction of C-terminal His-tagged *Gt*FlhG^K215E^ | (5) |
| pAD217 | Heterologous overproduction of N-terminal His-tagged *Gt*FliY | *This study* |
| pAD251 | Heterologous overproduction of GST-*Gt*FliY-N36 | *This study* |
| pAD306 | Insertion of *gpsB-mScarlet* into the *amyE*-locus under control of Phyper-spank | *This study* |
| pAD323 | Integration vector to fuse FliM to mNeonGreen under control of a xylose-inducible promotor | *This study* |
| pAD321 | Integration vector to fuse FlhG to mNeonGreen under control of a xylose-inducible promotor | *This study* |
| pAD129 | Markerless deletion of MTS of *flhG* (aa 279-296) | *This study* |
| pJPF007 | Markerless mutation of *flhG* D65A | *This study* |
| pJPF006 | Markerless mutation of *flhG* Q8A | *This study* |
| pAD255 | Insertion of *flhG* into the *amyE*-locus under control of Phyper-spank | *This study* |
| pAD254 | Insertion of *gpsB* into the *amyE*-locus under control of Phyper-spank | *This study* |
| pFA028 | Markerless Mutation of *hag*^T209C^ | *This study* |
| pAD228 | Markerless deletion of *ponA* | *This study* |
| pGBKT7-FlhG | plasmid expressing FlhG fused to the Gal4 DNA-binding domain | *This study* |
| pDK12897 | plasmid expressing GpsB fused to Gal4 activation domain | *This study* |
| pDK12898 | plasmid expressing amino acids 26 – 98 of GpsB fused to Gal4 activation domain | *This study* |
| pDK12899 | plasmid expressing amino acids 50 – 98 of GpsB fused to Gal4 activation domain | *This study* |
| pDK12900 | plasmid expressing amino acids 61 – 98 of GpsB fused to Gal4 activation domain | *This study* |
| pDK12901 | plasmid expressing amino acids 69 – 98 of GpsB fused to Gal4 activation domain | *This study* |
| pDK12902 | plasmid expressing amino acids 1 - 68 of GpsB fused to Gal4 activation domain | *This study* |

**Supplementary Table S4. Primers used in this study**

| **Primer** | **Sequence 5’ -> 3’** | **Purpose** |
| --- | --- | --- |
| PB11Flk1f | catgaattctgaagcaatcattgccgaatatggccg | 5’ flank for mutation of *flgE*^T123C^ |
| PB11Flk1r | ctgtaaccagtgttccacaattatctaaatagaagttc | 5’ flank for mutation of *flgE*^T123C^ |
| PB11Flk2f | gaacttctatttagataattgtggaacactggttacag | 3’ flank for mutation of *flgE*^T123C^ |
| PB11Flk2r | catggatccttcttaaaggtttcttttcccttgtctc | 3’ flank for mutation of *flgE*^T123C^ |
| AD176 | ttaaggtctcggatccctaggtgatttttcagcatatcaatct | 5’ flank for deletion of *flhG* |
| AD177 | ttaaggtctcatcaaactcgagtcatctgcacagcatcct | 5’ flank for deletion of *flhG* |
| AD178 | ttaaggtctcctcgagtttgattcgtgtgcttgtagtt | 3’ flank for deletion of *flhG* |
| AD179 | ttaaggtctcgaattctatgacggcttggtgtgt | 3’ flank for deletion of *flhG* |
| AD189 | ttaaggtctcggatccagcacgttcactactttcca | 5’ flank for deletion of *gpsB* |
| AD190 | ttaaggtctcgcgtgactttttcacctcgtatcgtgaa | 5’ flank for deletion of *gpsB* |
| AD191 | ttaaggtctctcacgcttgaaaaaaatggc | 3’ flank for deletion of *gpsB* |
| AD192 | ttaaggtctcgaattcaatgagcgccagttaatcg | 3’ flank for deletion of *gpsB* |
| AD64 | ttaaggtctcccatgggccagatgaacagatatgacca | Fwd *Bs flhG* |
| AD65 | ttaaggtctcctcgagttaagccctcctcattaaaaaagaagat | Rev *Bs flhG* |
| AD264 | ttaaggtctcccatgggctcagccaatcaggtaaaactg | Fwd *Gt gpsB* |
| AD269 | ttaaggtctcctcgagctattcatacagcttccggc | Rev *Gt gpsB* |
| AD256 | ttaaggtctcccatgggcgtgaaagatcaagcagaacagc | Fwd *Gt flhG* |
| AD257 | ttaaggtctcctcgagcctttctaggaaaagttggcg | Rev *Gt flhG* |
| AD295 | ttaaggtctcctcgagttaactcgcccgctcc | Rev *Gt flhG* ΔMTS |
| AD292 | ttaaggtctcccatgggctctggtgaatatcgttctcgt | Fwd *Gt ponA* |
| AD294 | ttaaggtctcctcgagttacgatagtccttttttctttttcgc | Rev *Gt ponA* |
| AD271 | ttaaggtctcctcgagcgtcgttcctgtcgg | Rev *Gt gpsB*-NTD |
| AD267 | ttaaggtctcccatgggcacgaactacgatattttgcagc | Fwd *Gt gpsB*-CTD |
| JSS07 | cgtatgccatgatggaatatatgcacgctg | Fwd *Gt flhG*^K177E^ |
| JSS08 | cagcgtgcatatattccatcatggcatacg | Rev *Gt flhG*^K177E^ |
| JSS013 | ggtatgaagtttttgaggagctgaagcacgtcaccg | Fwd *Gt flhG*^R207E^ |
| JSS014 | cggtgacgtgcttcagctcctcaaaaacttcatacc | Rev *Gt flhG*^R207E^ |
| JSS019 | cacgtcaccggtcggtctttaaacaaagatattgcg | Fwd *Gt flhGF*^215S^ |
| JSS020 | cgcaatatctttgtttaaagaccgaccggtgacgtg | Rev *Gt flhGF*^215S^ |
| AD298 | ttaaggtctcccatgggcatgaatgatggaatgttgtcg | Fwd *Gt fliY* |
| AD299 | ttaaggtctcctcgagttttagccgttttaaccgatcgt | Rev *Gt fliY* |
| AD351 | ttaaggtctcctcgagttaactcatttccccagcagaaa | Rev *Gt fliY*-N36 |
| AD353 | ttaagtcgacagtaaggaggattttagaatgcttgctgataaagtaaagct | Fwd *Bs gpsB* for pDR111 |
| AD354 | ttaagcatgctcaatcataaagcttgctgcc | Rev *Bs gpsB* for pDR111 |
| AD484 | ttaaggtctcgaattcggtaattataaagaagcttggcagt | Fwd *Bs fliM* for pSG1164 |
| AD485 | ttaaggtctcggatccttctccatcttgttcacctcttat | Rev *Bs fliM* for pSG1164 |
| AD482 | ttaaggtctcgaattcgccaatgaactttctcatgc | Fwd *Bs flhG* for pSG1164 |
| AD483 | ttaaggtctcggatccagccctcctcattaaaaaagaag | Rev *Bs flhG* for pSG1164 |
| AD194 | ttaaggtctcggatccatgaacagatatgaccaagcag | Fwd *Bs flhG* ΔMTS |
| AD195 | ttaaggtctcccatggcggcttggtgtgtcatggttatc | Rev *Bs flhG* ΔMTS |
| JPF001 | atggtaggtctcggatcccagcatatcaatctgtacttcctgag | Fwd 5’ flank for *Bs flhG* mutation |
| JPF002 | atggtaggtctcgaattcggcttggtgtgtcatggttatc | Rev 3’ flank for *Bs flhG* mutation |
| JPF003 | atggtaggtctcgacgcagcagcaactttacgggcg | Fwd 3’ flank for *Bs flhG*^Q8A^ |
| JPF004 | atggtaggtctctgcgtcatatctgttcatctgcacagc | Rev 5’ flank for *Bs flhG*^Q8A^ |
| JPF005 | atggtaggtctccttgcaatcgggatggggaacattg | Fwd 3’ flank for *Bs flhG*^D65A^ |
| JPF006 | atggtaggtctctgcaaggtcgatgagcagcaccttcttac | Rev 5’ flank for *Bs flhG*^D65A^ |

**Supplementary References.**

1. M. A. Konkol, K. M. Blair, D. B. Kearns, Plasmid-encoded ComI inhibits competence in the ancestral 3610 strain of Bacillus subtilis. *J Bacteriol* **195**, 4085-4093 (2013).

2. J. R. Huth *et al.*, Design of an expression system for detecting folded protein domains and mapping macromolecular interactions by NMR. *Protein Sci* **6**, 2359-2364 (1997).

3. M. Arnaud, A. Chastanet, M. Debarbouille, New vector for efficient allelic replacement in naturally nontransformable, low-GC-content, gram-positive bacteria. *Appl Environ Microbiol* **70**, 6887-6891 (2004).

4. M. Strach, F. Koch, S. Fiedler, K. Liebeton, P. L. Graumann, Protein secretion zones during overexpression of amylase within the Gram-positive cell wall. *BMC Biol* **21**, 206 (2023).

5. J. S. Schuhmacher *et al.*, MinD-like ATPase FlhG effects location and number of bacterial flagella during C-ring assembly. *Proc Natl Acad Sci U S A* **112**, 3092-3097 (2015).
